# Divergent functional isoforms drive niche specialisation for nutrient acquisition and use in rumen microbiome

**DOI:** 10.1038/ismej.2016.172

**Published:** 2017-01-13

**Authors:** Francesco Rubino, Ciara Carberry, Sinéad M Waters, David Kenny, Matthew S McCabe, Christopher J Creevey

**Affiliations:** 1 Institute of Biological, Environmental and Rural Sciences (IBERS), Aberystwyth University, Aberystwyth, UK; 2 Animal and Bioscience Research Department, Teagasc, Grange, Dunsany, Co., Meath, Ireland; 3 School of Agriculture, University College Dublin, Dublin, Ireland

## Abstract

Many microbes in complex competitive environments share genes for acquiring and utilising nutrients, questioning whether niche specialisation exists and if so, how it is maintained. We investigated the genomic signatures of niche specialisation in the rumen microbiome, a highly competitive, anaerobic environment, with limited nutrient availability determined by the biomass consumed by the host. We generated individual metagenomic libraries from 14 cows fed an *ad libitum* diet of grass silage and calculated functional isoform diversity for each microbial gene identified. The animal replicates were used to calculate confidence intervals to test for differences in diversity of functional isoforms between microbes that may drive niche specialisation. We identified 153 genes with significant differences in functional isoform diversity between the two most abundant bacterial genera in the rumen (*Prevotella* and *Clostridium).* We found *Prevotella* possesses a more diverse range of isoforms capable of degrading hemicellulose, whereas *Clostridium* for cellulose. Furthermore, significant differences were observed in key metabolic processes indicating that isoform diversity plays an important role in maintaining their niche specialisation. The methods presented represent a novel approach for untangling complex interactions between microorganisms in natural environments and have resulted in an expanded catalogue of gene targets central to rumen cellulosic biomass degradation.

## Introduction

Recent metagenomic sequencing of natural environments has revealed that microbial communities act in different trophic levels (Lauro *et al.*, 2009), exhibit successional change (Koenig *et al.*, 2011; Teeling *et al.*, 2012; Huws *et al.*, 2016) and engage in fierce competition when resources are limited (Weckx *et al.*, 2011). This suggests that adaptation to specific niches must also occur in microbial communities, however demonstrating this in natural communities remains a challenge (Marco, 2008).

It has been proposed that radical niche diversification in prokaryotes may primarily occur through horizontal acquisition of novel genes (Jain *et al.*, 2003; Toft and Andersson, 2010). Furthermore, the high frequency of horizontal gene transfer detected among bacteria (Gogarten *et al.*, 1999; Creevey *et al.*, 2004; Nelson-Sathi *et al.*, 2014), even among ‘core’ genes (Creevey *et al.*, 2011), suggests that horizontal gene transfer may also drive niche differentiation even within species (Coleman *et al.*, 2006).

In contrast, microbial community profiling using 16S ribosomal RNA has revealed core communities can be defined for many environments (Turnbaugh *et al.*, 2009; Lundberg *et al.*, 2012; Creevey *et al.*, 2014; Otani *et al.*, 2014), indicating that niche specialisation of specific groups of organisms is somehow maintained even under the continued influence of horizontal gene transfer. One explanation may be that the horizontal acquisition of a single isoform of a novel gene is not enough to maintain a competitive advantage in fluctuating environmental conditions where a range of isoforms may be required to, for instance, maintain enzymatic activity (Stewart, 1976; Nevo, 2001).

The rumen microbiome of sheep and cattle is a case in point; temperature and pH fluctuate daily in response to ambient conditions and the biomass consumed (Mishra *et al.*, 1970). As a potential source of industrially important enzymes (Hess *et al.*, 2011; Qi *et al.*, 2011), antimicrobial agents (Russell and Mantovani, 2002) and greenhouse gases (Qi *et al.*, 2011), understanding how the microbiome maintains robustness to environmental fluctuations and how this may be manipulated is an important goal.

Identifying gene isoforms from metagenomic sequence data remains a difficult challenge (Scholz *et al.*, 2012), as assembly approaches vastly oversimplify the true microbial diversity present (Temperton and Giovannoni, 2012). Recently, approaches that involve mapping metagenomic sequence reads, (which preserve the lost variability), to sequenced microbial genomes have attempted to address this by identifying single-nucleotide polymorphisms (SNPs) representing variation in the microbial population (Williams and Oleksiak, 2011; Schloissnig *et al.*, 2013). Using methods adapted from traditional dn/ds calculations of adaptive evolution between species (Creevey and McInerney, 2003; Loughran *et al.*, 2012; Baldwin *et al.*, 2014) they calculated the genomic variation in the human gut microbiome based on the ratio of the rates of occurrence of SNPs that either cause a mutation in the translated amino acid sequence (non-synonymous mutations; pN) or preserve the translated amino acid sequence (synonymous mutations; pS; Schloissnig *et al.*, 2013). The population with a higher pN/pS ratio for any function likely reflects the possession of greater numbers of distinct functional isoforms of that gene (Schloissnig *et al.*, 2013). Furthermore, as this calculation uses the rate of synonymous substitutions as a baseline, it is robust to differences in the size of the populations compared.

We have adapted this approach to examine the rates of pN/pS for all shared functions between the two most abundant genera of bacteria in the rumen. Applied to metagenomic samples from 14 animals maintained as a single herd and provided with the same diet, we were able to estimate confidence intervals for the pN/pS ratios calculated. We then investigated whether differences in the abundance of functional isoforms existed between the two microbial populations and therefore could possibly drive niche specialisation.

## Materials and methods

### The animal model

The animal model on which this work is based has been described previously (Carberry *et al.*, 2012) and was part of a larger study designed to examine the physiological control of energetic efficiency in growing beef heifers (Kelly *et al.*, 2010). Our analysis centred on samples taken during this study from 14 Limousin x Friesian heifers following a 44-day period of *ad libitum* access to a high-forage diet of grass silage. Samples of rumen fluid were collected using a transesophageal sampling device (FLORA rumen scoop; Guelph, ON, Canada). Subsequently, a 20-ml aliquot was transferred using a pipette and sterilised tip into a separate labelled sterilised container, immediately frozen in liquid nitrogen, and stored at 80 °C until processing (Carberry *et al.*, 2012).

### Sampling, metagenomic library preparation and sequencing

DNA was extracted from rumen fluid samples using the repeat bead beating and column purification method of Yu and Morrison (2004). DNA was fragmented to an average size of 300 bp using a Bioruptor (Diagenode, Seraing, Belgium). The NEBNext End Repair Module was used to blunt end the fragments and purification of the reaction was performed using a QIAquick PCR purification kit (Qiagen, Hilden, Germany). The NEBNext dA-Tailing Module was used to adenylate the blunt-ended fragments and purification of the reaction was performed using a QIAquick PCR purification kit (Qiagen). Illumina standard paired end barcoded adapters were ligated onto the adenylated fragments using the Quick Ligation Kit (NEB, Ipswich, MA, USA) and purification was performed using a QIAquick PCR purification kit (Qiagen). Adapter-ligated fragments were then size selected (average insert size of 280 bp) by electrophoresis on an agarose gel, excision of a 2 mm gel slice and extraction of DNA from the agarose using the QIAquick gel extraction kit (Qiagen). PCR enrichment (12 cycles) of the library was performed using Illumina PCR Paired End Primers 1.0 and 2.0 and Phusion High-Fidelity PCR Kit (Finnzymes, Waltham, MA, USA). The library size and absence of adapter dimers were determined with a DNA1000 chip on an Agilent 2100 Bioanalyzer (Agilent, Santa Clara, CA, USA). Libraries were pooled in equimolar amounts and sequenced on an Illumina GAIIx using a standard paired end 300 cycle sequencing kit.

### Assembly

The read length for this data set was 160 bp and after quality control 15 bp were trimmed from both 5′ the 3′ ends. The trimmed sequences from all 14 samples were used to create a combined metagenome assembly with SGA, version 0.9.18 (Simpson and Durbin, 2012). First, pre-processing of the reads was carried out using the preprocess module of SGA with the –phred64 flag, with settings -p1 for paired-end libraries and –permute-ambiguous to treat ambiguities as all possible combinations of characters. Second, the computationally and memory intensive indexing step was carried out using the index module of SGA with the default options. Finally, the pre-processed and indexed reads were used as input to the metagenome module of SGA to carry out the assembly with the default options, except for the -k 51 flag, for a k-mer size of 51. The resulting assembly contained all contigs ⩾200 bp in length. The raw sequence data can be accessed at the European Nucleotide Archive (http://www.ebi.ac.uk/ena) using the study ID: ERP009980.

### Gene and taxonomy prediction

Gene predictions were carried out on the assembly using HMMER version 3.0 (Eddy, 2011). To do this, HMM profiles of known genes were generated for each gene family from within each group of organisms from the rumen (Creevey *et al.*, 2014). Genes were identified from the Kegg Orthology (Kanehisa *et al.*, 2012) database and the corresponding sequences downloaded from Uniprot (The Uniprot Consortium, 2012) using the download_profiles script of the MGKit package (Rubino and Creevey, 2014; version 0.1.13). This script automates the retrieval of all ortholog sequences available for all combinations of genes and taxa of interest. Only combinations of genes and taxa having at least two sequences in Uniprot were used (a list of taxa is available in Supplementary Table S1). Alignments were created for each gene family from a group of organisms using Clustal Omega (Sievers *et al.*, 2011) version 1.1.0 and converted into HMMER profiles using the HMMbuild command. These profiles were used as input to HMMsearch for comparison against the contigs from the assembly, which were translated into all six reading frames using the translate_seq script in MGKit.

### Converting results to annotations

Matches of contigs to the HMM profiles were converted into GFF format and combined with annotation information from KEGG using the hmmer2gff script of MGKit. They were then filtered for low-quality predictions and only matching regions in the assembly with an e-value threshold of 0.05 were retained. Furthermore, using the filter_gff script of MGKit any annotation whose length was covering <40% of the average length of the HMM profile it matched was discarded (-f option). All annotated regions that overlapped for more than 80% of their length were discarded, except the one having the lowest e-value, (-l option). Taxonomic assignments of the annotated regions were carried out using BLAST+ version 2.2.25 (Camacho *et al.*, 2009), against a local copy of the *nr* protein database (May 2013; options -outfmt 6). The best match with a bit score >60 was used as simulations indicated that when using this cut-off hits were correctly assigned at the genus level 95% of the time (Supplementary File 1).

### Alignment

All 14 sample reads were individually aligned to the final assembly, using Bowtie2 (Langmead and Salzberg, 2012) version 2.0.5 (options: -N 1). Possible PCR and optical duplicate reads were identified using Picard Tools (Team, 2009) version 1.58. Abundance of reads mapping to each annotation was calculated with htseq-count from the HTSeq package (Anders *et al.*, 2015) version 0.5.4p3 (options: -s no -t gene -i ko_idx –q).

### SNPs

SNPs were called from each of the alignments separately using the GATK pipeline (McKenna *et al.*, 2010), version 2.1.8. with the UnifiedGenotyper tool (options: -dcov 50 for 4 × coverage alignment). The resulting VCF files were analysed to identify for each SNP whether it was synonymous using SNPDat version 1.0.4 (Doran and Creevey, 2013). All VCF files were then combined for analysis using the CombineVariants command of GATK (options: -genotypeMergeOptions UNIQUIFY to make all sample genotypes unique by file name). The output of SNPDat and the merged VCF file were combined using the snp_parser script of MGKit. The retained SNPs had a minimum allele frequency of 0.01, a minimum Phred quality score of 30 and supported by a minimum of four reads across all the samples.

### Calculation of rates of pN/pS

The pN/pS measure (Schloissnig *et al.*, 2013) is the ratio of two quantities, pN and pS. The former is the ratio between the number of non-synonymous amino acid changes to those expected, whereas the latter describes the same ratio for synonymous changes. The expected number of changes is calculated in a similar way as in dn/ds calculations, using a neutral model to determine the proportion of either non-synonymous or synonymous SNPs that would be expected given the reference sequence for the gene (Li, 1993). The MGKit package provides routines for these calculations on a per-isoform basis, but they can be extended to different levels of specificity, for example, using genus instead of species, or enzymes grouped into different EC levels such as 3.4.1.3 or EC 3.4.-. This involves concatenating isoforms belonging to the specific taxonomic and functional level before performing the calculation. When calculating the rates of pN/pS of individual functions within taxonomic groups, if multiple genes from the assembly were annotated with the same function and taxonomy, they were concatenated and the rates of pN/pS calculated across all the concatenated sequences.

### Statistical analysis of functional divergence

**Figure 1:**
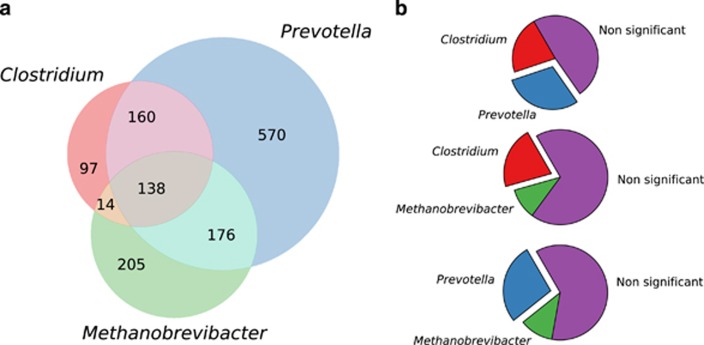
Gene counts from the three most abundant genera in the rumen samples. (**a**) Venn diagram showing the overlap of genes found in at least three samples between the *Prevotella*, *Clostridium* and *Metahonbrevibacter*. (**b**) The number of genes found in at least two genera, indicating the proportion that had a significantly higher pN pS rates in one group or the other (b1) between *Clostridium* and *Prevotella*, (b2) between *Clostridium* and *Methanobrevibacter* and (b3) between *Methanobrevibacter* and *Prevotella.*

Pairwise comparisons of the distribution of pN/pS calculations for genes were carried out between taxa using a two-sample Wilcoxon rank-sum test as implemented in the Scipy package (van der Walt *et al.*, 2011) version 0.15.1. In this analysis, pairwise statistical tests were carried out between all genes shared between *Methanobrevibacter*, *Clostridium* and *Prevotella* (Figure 1). All *P*-values were corrected for multiple testing using the Benjamini–Hochberg (Benjamini and Hochberg, 1995) method as implemented in R (R Core Team, 2014), using a significance threshold for the corrected value of 0.1.

### Enzyme Commission Classification

Functional diversity was also estimated for all predicted genes from the metagenomic data that had a mapping to an enzyme commission classification (EC). We concatenated all the genes from same second-level EC classification (that is, EC: 1.1.-, 1.2.- and so on), only including a predicted gene if it had been found in both *Prevotella* and *Clostridium* in at least seven samples. A single pN/pS value was calculated for each sample, representing the diversity of all the genes for any particular EC number and the distributions of the pN/pS values across samples were compared between *Clostridium* and *Prevotella*. A Wilcoxon rank-sum test as implemented in R was used to test whether the distribution of diversity estimates for any EC number was significantly different between the two groups and the *P*-values were corrected as previously described.

### Over-representation analysis

The EC classifications that were found to have significantly different pN/pS values between *Prevotella* and *Clostridium* were used for an over-representation analysis. The significantly different EC numbers were used as foreground for the analysis, whereas the rest of the all EC numbers *Prevotella* and *Clostridium* had in common, were used as background. The files were then analysed in R, with the GOSeq package (Young *et al.*, 2010) to perform the test and adjusting the *P*-values with the Benjamini–Hochberg method.

### Coverage calculation

As the length of the reads was fixed at 130 bp for this data set, the coverage formula used is the following: 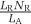, where *L*_R_ is the length of the reads aligned, *N*_R_ is the number of reads mapping to the specific region and *L*_A_ is the length of the annotation for which coverage is calculated.

All MGKit scripts used are provided as HTML files in Supplementary Files 1–7. MGKit is available with tutorials under the GNU General Public License at https://bitbucket.org/setsuna80/mgkit/.

## Results

### General assembly results

The average number of reads generated per sample was 11.7 M (min 8.6 M, max 14.0 M) totalling 164.3 M reads in total. The total number of assembled contigs with a minimum size of 200 bp was 1  421  712 with an average length of 374 bp (max 62  508 bp) and an N50 of 364. The number of predicted genes passing filters from the assembly was 106  475 with 70  525 having at least 4 × coverage of aligned reads in at least one sample (30  970 in at least 3 samples, 2665 across all 14 samples). The average gene coverage per sample was 8.7 × (min 4 ×, max 1264 ×). More details are provided in Table 1.

### Taxonomic and functional annotation

Following taxonomic assignments, counting only those genera with at least four observed genes and with an average coverage among all samples ⩾4 × resulted in 127 bacterial, 20 archaeal, 5 fungal and 21 protozoal genera remaining (Supplementary Table S3). Functional annotation of these genes revealed that 20.12% were involved in information storage and processing, 33.96% were involved in metabolism, 17.71% were involved in cellular processes and signalling and 28.21% were poorly characterised (Supplementary Figure S1).

This revealed that the genus with the largest number of genes in our data set was the bacterial genus *Prevotella* (1044 genes), followed by the archaeal genus *Methanobrevibacter* (533 genes) and then the bacterial genus *Clostridium* (409 genes). When only considering those with matches to gene families from eggNOG (Powell *et al.*, 2012), 16.03% were from *Prevotella*, 8.18% from *Methanobrevibacter* and 6.28% from *Clostridium* (Figure 1a). In the following analyses we concentrated on the two most abundant bacterial genera due to their specific roles in the colonisation of plant material in the rumen (Huws *et al.*, 2016).

### Diversity estimates

pN/pS values were calculated for all genes found in a minimum of three samples and with a minimum of 4 × coverage.

**Figure 2:**
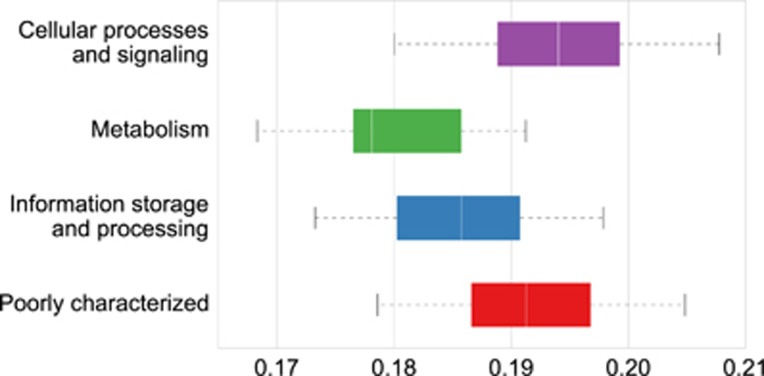
Functional categories variation among all genes with at least 4 × coverage. The *x* axis shows the pN/pS values and the *y* axis the functional categories.

The calculated pN/pS values ranged from 27.16 in *gpmI* (Kegg ID: K15633) from *Prevotella*, which is involved in carbohydrate and lipid metabolism, to many genes (59.80% of the total) with a pN/pS value of zero. When assessed by function using eggNOG the category ‘cellular processes and signalling’ had the highest average pN/pS (0.19), and ‘metabolism’ had the lowest (0.18; Figure 2). Comparison of the pN/pS values of the genes shared by the two most abundant genera of bacteria (*Prevotella* and *Clostridium*) revealed that 88 and 65 had significantly higher pN/pS values (adjusted *P*<0.1) in *Prevotella* and *Clostridium*, respectively (Figure 1b).

### KEGG pathways

**Figure 3:**
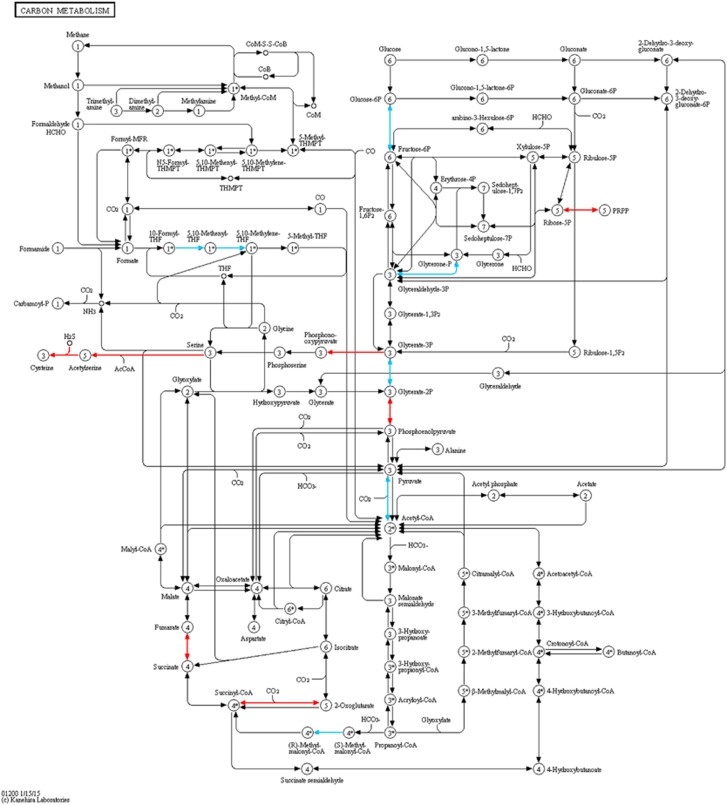
Genes marked in blue and red are significantly different between *Prevotella* and *Clostridium* and are part of the carbon metabolism pathway in KEGG. Genes in blue are significantly different between the two genera and have a higher average pN/pS in *Prevotella*. Genes in red have a higher average in *Clostridium.*

**Figure 4:**
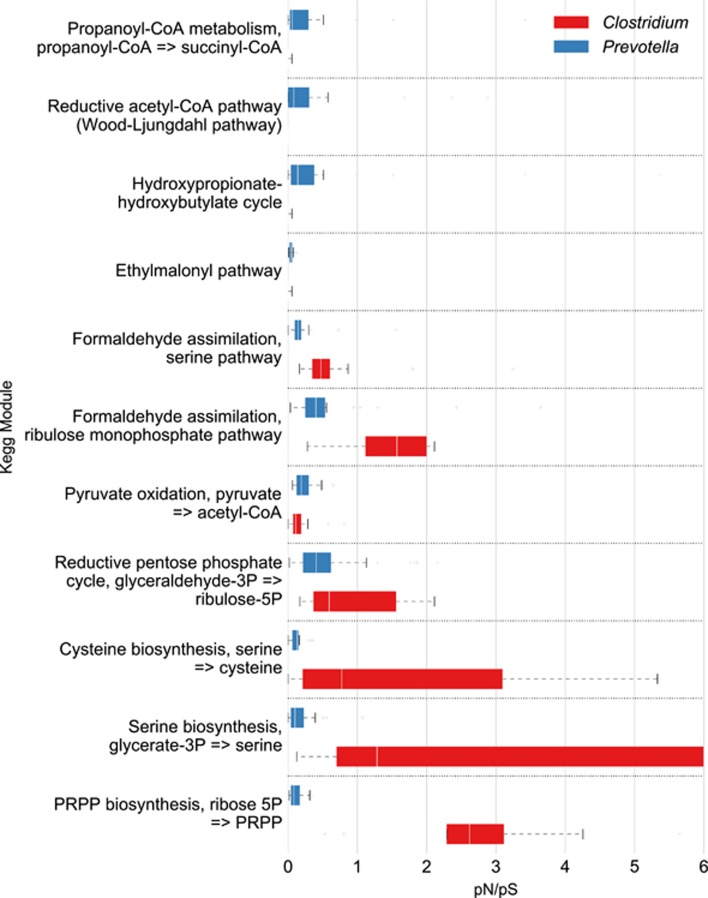
Variation for module of the carbon metabolism that were found to be significantly different. Red *(Clostridium*), blue (*Prevotella*).

Visual comparison of the rates of pN/pS superimposed on various KEGG metabolic pathways, identified the ‘Carbon metabolism’ pathway, as showing clearly connected sub-components of the pathways where the genes involved either had a significantly higher pN/pS in *Prevotella* compared with *Clostridium* or vice versa (Figure 3). To investigate this in more detail we broke the ‘Carbon metabolism pathways’ into its constituent sub-components (called modules in KEGG). Overall pN/pS values were calculated from each of the 14 samples for each pathway module following concatenation of each of the genes in that module. The distribution of pN/pS values from the multiple samples was used to test if a significant difference existed in their functional diversity for any module between *Prevotella* and *Clostridium*. This identified the following modules as being significantly different (*P*<0.1 after correction) between *Prevotella* and *Clostridium*: ‘Pyruvate oxidation’, ‘Hydroxypropionate-hydroxybutyrate’, ‘Phosphate acetyltransferase-acetate kinase pathway, acetyl-CoA => acetate’ were more diverse in *Prevotella;* whereas ‘PRPP biosynthesis ribose 5P’, ‘Cysteine biosynthesis—serine’, ‘Formaldehyde assimilation—ribulose monophosphate pathway’, ‘Formaldehyde assimilation—serine pathway’ were found more diverse in *Clostridium* (Figure 4; Supplementary Table S4).

### Enzyme Commission Classification

Twenty-one EC showed significantly divergent rates (*P*<0.05) between *Prevotella* and *Clostridium*.

The EC with significantly higher diversity in *Prevotella* included oxidoreductases acting on the CH-OH group of donors (EC 1.1.-), the aldehyde or oxo group of donors (EC 1.2.-), and on the CH-NH2 group of donors (EC 1.4.-); Hydrolases acting on peptide bonds (EC 3.4.-); General lyases (EC 4.99.-) and the intramolecular oxidoreductase category of isomerases (EC 5.3.-) (Supplementary Figure S2).

The EC with a significantly higher pN/pS distribution in *Clostridium* included: oxidoreductases acting on the CH-CH group of donors (EC 1.3.-) and on NADH or NAPH (EC 1.6.-); transferases transferring aldehyde or ketonic groups (EC 2.2.-), acyltransferases (EC 2.3.-), glycosyltransferases (EC 2.4.-) and transferring phosphorus-containing groups (EC 2.7.-); hydrolases, specifically glycosylases (EC 3.2.-), those acting on carbon–nitrogen bonds, other than peptide bonds (EC 3.5.-), and those acting on carbon–carbon bonds (EC 3.7.-); carbon–carbon lyases (EC 4.1.-); racemases and epimerases (EC 5.1.-) and *cis*-*trans*-isomerases (EC 5.2.-) from isomerases, and finally, ligases forming carbon–oxygen bonds (EC 6.1.-), carbon–sulfur bonds (EC 6.2.-) and nitrogen–metal bonds (EC 6.6.-; Supplementary Figure S3).

Supporting this, genes found to be significantly divergent between *Prevotella* and *Clostridium* were also statistically overrepresented in 14 of the EC already identified. In particular, the EC 1.1.-, 1.2.-, 1.4.-, 5.3.- and 3.4.- were significantly overrepresented in the set of genes that were more diverse in *Prevotella* (*P*<0.001) and the EC 1.3.-, 2.3.-, 2.4.-, 2.7.-, 3.2.-, 3.5.-, 4.1.- 5.1. and 6.1.- were significantly overrepresented in the set of genes that were more diverse in *Clostridium* (*P*<0.05; Supplementary Table S2).

## Discussion

### Taxonomic composition

The fact that there are very few fully sequenced rumen microbial genomes available have likely inflated the estimates of the number of genera we reported (Supplementary Table S3), as when the genome is not in the database, the same gene from one of the most closely related species is likely to be matched instead. However if only those genera with a minimum four genes identified are considered, then 127 bacterial and 20 archaeal genera remain, which is similar to previous 16S-based estimates of both bacteria (Creevey *et al.*, 2014) and archaea (Janssen and Kirs, 2008).

However, the most interesting result from the taxonomic identification was from the most abundant groups. We found that *Methanobrevibacter* genes were more abundant than genes from *Clostridium* and second only to the number of genes found from *Prevotella* (Supplementary Table S3). These findings are contrary to estimates that would place the abundance of archaea well below that of these two groups. Previous estimates of archaeal abundance from the rumen using culture-based approaches tend to be at around 4% (Yanagita *et al.*, 2000), with numbers from metatranscriptomics suggesting that archaeal messenger RNA represents somewhere between 0.1 (Qi *et al.*, 2011) and 1% (Poulsen *et al.*, 2013) of the total messenger RNA actively transcribed. We estimate from our samples that the minimum proportion of *Methanobrevibacter* genes in the rumen metagenome is 9.02%, following *Prevotella* at 17.66% and followed by *Clostridium* at 6.92%. The differences observed between our current and previous estimates are likely due to differences in the technologies used (Janssen and Kirs, 2008; Steinberg and Regan, 2008). For instance, biases in the primers used for ribosomal RNA amplification in archaea can under-represent certain groups and because of differences in the primers used, the comparison of abundances of archaea and bacteria is nontrivial (Tymensen and McAllister, 2012).

**Figure 5:**
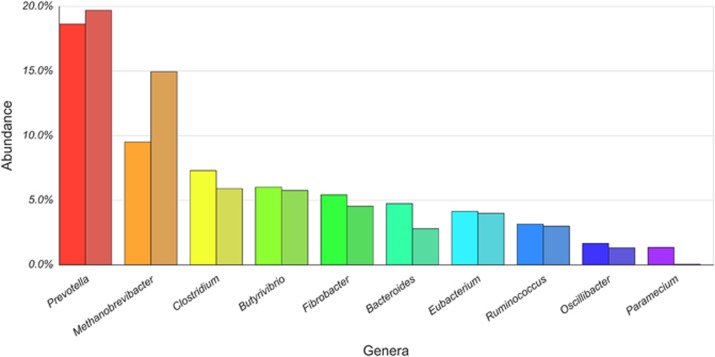
Number of genes found in each genus. For each genus the proportion of number of genes is plotted in a different colour. Each genus has two bars, the left one is the proportion of genes found and the right bar the proportion of genes scaled by the average genome size of the genus. A full colour version of this figure is available at the *ISME Journal* online.

To our knowledge it has not previously been suggested that the *Methanobrevibacter* genes could make up a larger component of the rumen metagenome than *Clostridium*. Scaling these numbers according to average genome sizes using sequenced organisms from these groups actually increases this trend (Supplementary Table S3; Figure 5). However our estimates of the proportion of archaeal genes in the rumen metagenome from *Methanobrevibacter* (76.47%) and the relative proportions of genes from the major groups of bacteria are similar to those that were previously reported (Janssen and Kirs, 2008; Creevey *et al.*, 2014), suggesting no sampling bias.

### Diversity estimates

**Figure 6:**
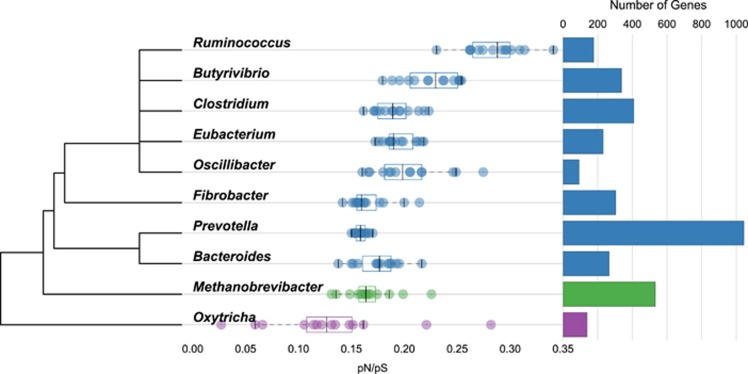
Variation in pN/pS calculated for all genes assigned to the genera representing the top 55% of all genes identified. Each box plot represents the distribution of pN/pS values calculated across the samples. The values for the individual samples are represented by the individual data points superimposed on the boxplots. For each genus, all genes from the same sample were concatenated and pN/pS values calculated, with the total number of (full and partially reconstructed) genes used indicated by the histogram on the right. Colours represent the group to which the genus belongs: bacteria (blue), archaea (green) and protozoa (purple).

We found that levels of adaptive variation (pN/pS) vary widely between microbial genera from the rumen (Figure 6) similar to that found between human gut microbes (Schloissnig *et al.*, 2013) and that this extends to functional classes of genes (Figure 2; Supplementary Figures S2 and S3) and also for the same genes from different genera.

However, the level of variation in these calculations between samples was related to the abundance of each genus in the rumen microbiome (Figure 6), and therefore the depth of sequencing achieved for each. Although the low variance in the calculations across samples of pN/pS for *Prevotella* (0.000033) and *Clostridium* (0.000363) provided enough confidence to further investigate these groups, greater depth of sequencing will be necessary for other genera. Although the pN/pS variance in the archaea *Methanobrevibacter* is comparable to both *Prevotella* and *Clostridium*, we focussed on comparisons between the latter two bacterial genera, which have been shown to directly compete for access to the plant fibre (Huws *et al.*, 2016). The specific ecological niche of methanogens in the rumen is quite distinct and they do not directly compete for access to the plant fibre material. They utilise hydrogen and carbon dioxide as an energy and carbon source, respectively, and produce methane as a by-product (Carberry *et al.*, 2014).

For *Prevotella* and *Clostridium*, we tested for significant differences in the pN/pS calculated between all shared genes, testing the hypothesis that this reflected functional importance of those genes to the organism. In this scenario a gene with significantly higher values of pN/pS in one genus compared with the other indicates higher levels of functional isoforms within its population. We hypothesise that these isoforms may allow important functions for that organism to be robust to fluctuations in the environmental conditions (or substrates) within the rumen. Functional analysis of these genes should reflect this, allowing construction of a profile of functions for each that reflects their roles in the rumen.

### Metabolic adaptations in *Prevotella*

We found that *Prevotella* possess more diverse functional isoforms than *Clostridium* in genes involved in specific metabolic processes. For instance, pyruvate oxidation (from the carbon metabolism pathway) was significantly more diverse in *Prevotella* (average of 1.50 versus 0.16) as was the enzyme class 1.2.-, which contains pyruvate oxidase. Interestingly both the substrates for this pathway (pyruvate) and its product (acetyl-CoA) are central to fermentative processes and the reactions involved have been implicated in anaerobic fermentation of a diverse selection of compounds by *Prevotella* (Takahashi and Yamada, 2000; Purushe *et al.*, 2010) and other species (Abdel-Hamid *et al.*, 2001). Studies of pyruvate oxidase in *Escherichia* *coli* have suggested that it is used preferentially at low growth rates (Abdel-Hamid *et al.*, 2001), indicating the intriguing possibility of an adaptation in *Prevotella* species in low nutrient conditions. Indeed, *Prevotella* have been identified as one of the primary colonisers of plant material consumed by the host, reaching its peak population within 1 h of incubation (Huws *et al.*, 2016). Therefore, a strategy that would allow them to maintain a sufficient population during nutrient scarcity that enables this rapid colonisation would be advantageous.

*Prevotella* also possessed significantly more functional diversity in the genes belonging to the hydroxypropionate-hydroxybutyrate cycle than *Clostridium* (average of 0.74 versus 0.01). This is involved in the conversion of carbon compounds in fatty acids (Ellis *et al.*, 2008; Servinsky *et al.*, 2012), and their higher diversity suggests *Prevotella* has a greater repertoire of enzymes involved in this process and supports previous reports that *Prevotella* species are among those (including *Clostridium* species) that play a role in biohydrogenation of dietary polyunsaturated fatty acids to saturated fatty acids (Huws *et al.*, 2011). This would suggest that fatty acids may be a resource for which these organisms compete and for which they have diversified niches.

Finally, when examined by their ECs, we found a significantly larger diversity of peptidases (EC 3.4) in *Prevotella* compared with *Clostridium* (average pN/pS 0.28 compared with 0.1) supporting previous reports of the importance of proteolysis as one of the main sources of metabolic energy for *Prevotella* (Wallace, 1996). This evidence suggests that *Prevotella* possess a greater diversity of functional isoforms than *Clostridium* that digest peptides, and may be related to the essential production of the volatile fatty acids propionate and butyrate used as nutrients by the host.

### Metabolic adaptations in *Clostridium*

We found that *Clostridium* possessed a significantly higher functional diversity than *Prevotella* for genes involved in a range of metabolic processes including cysteine biosynthesis (average 1.64 versus 0.13) and formaldehyde assimilation/serine pathway (average 0.75 versus 0.18). Interestingly, some *Clostridium* species from the rumen have been characterised as having a preferential utilisation for serine, threonine, cysteine, proline and glycine in their exponential growth phase (Flythe and Kagan, 2010; Fonknechten *et al.*, 2010). Our results suggest that this may be a specific niche specialisation by these species in the rumen.

Furthermore, the PRPP biosynthesis module that connects the non-oxidative branch of the pentose phosphate pathway to the histidine biosynthesis pathway (Nölling *et al.*, 2001) was also significantly more diverse in *Clostridium* than *Prevotella* (*P*<0.01, average of 2.76 versus 0.13). This supports reports that species of *Clostridium* may utilise histidine as part of a strategy to overcome nutrient limitation during the stationary growth phase (Fonknechten *et al.*, 2010).

Finally, the class of glycosylases to which the cellulosome enzymes from *Clostridium* species belong (EC 3.2; Ravachol *et al.*, 2014), was more functionally divergent in *Clostridium* than in *Prevotella*. This suggests *Clostridium* possess a greater range of diverse functional isoforms involved in catabolism of these polysaccharides than *Prevotella* and may provide an advantage in competitive conditions.

## Conclusion

**Figure 7:**
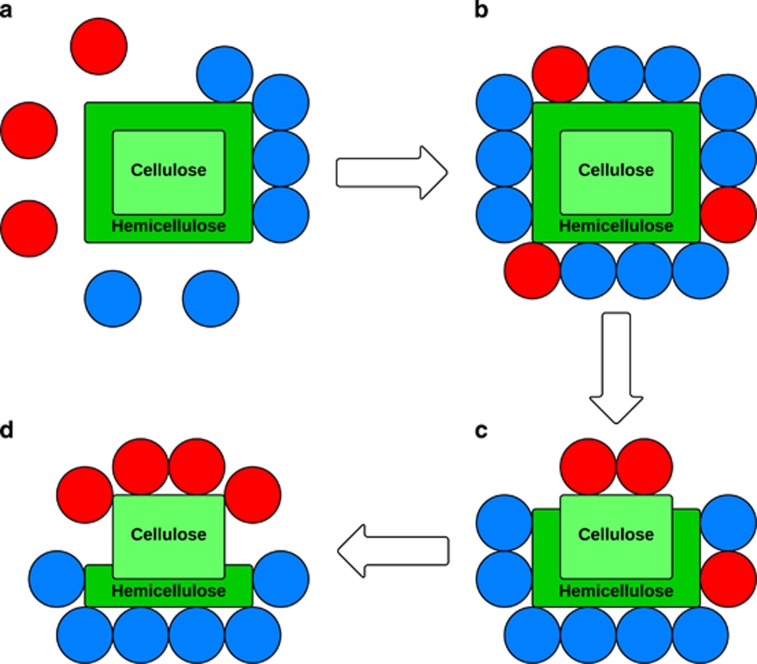
Cartoon illustration of colonisation of plant material in the rumen by *Prevotella* (blue) and *Clostridium* (red); plant fibre in green, composed by cellulose inside (bright green) and hemicellulose outside (dark green). Data suggest that the degradation process begins with *Prevotella* populating fibre faster than *Clostridium*, because its niche specialisations allowing more efficient action on hemicellulose (**a**) resulting in higher number of *Prevotella* observed in the early stages of plant fibre colonisation (**b**). However, as more cellulose becomes available (**c**) niche adaptations in *Clostridiu*m reverses this trend and increasing their numbers in the latter stages (**d**).

In this study we focussed on the most abundant bacterial genera in the rumen, showing how niche specialisation is driven by resource competition. This is exemplified by the development of differences in a range of isoforms in *Clostridium* and *Prevotella* for key genes that can provide a competitive advantage over the other. For instance, although both have the potential to degrade a complex matrix like plant fibre, they show significantly different adaptations relating to the structure of the plant material they colonise. In the rumen, colonisation of plant fibre is a multi-step process and it has been noted that the abundance of *Prevotella* peaks before *Clostridium* (Huws *et al.*, 2016). This has been hypothesised to relate to the degradation of the hemicellulose prior to cellulose in the plant. In this scenario, *Prevotella* would preferentially degrade hemicellulose, and when xylan compounds then become accessible, colonisation of the fibre surface is gradually taken over by *Clostridium* (Figure 7; Huws *et al.*, 2016). Our results indicate that this is likely the case as *Prevotella* has a more diverse range of isoforms to degrade the hemicellulose matrix formed of pectins (EC 5.3.-; Oxenbøll *et al.*, 2007) and peptides (EC 3.4.-), whereas *Clostridium* has a more diverse range of isoforms for degrading cellulose (EC 3.2.-). This coupled with their divergence for function in metabolic processes, indicate that isoform diversity plays an important role in maintaining their niche specialisation.

Our results demonstrate the potential power of evolutionary approaches in understanding microbial communities *in vivo*. Indeed, applied to less well-studied microbiomes, it should be possible to identify niche specialisation and generate novel hypotheses of the organisational structure and function of the constituents that could be tested *in vitro*.

## Figures and Tables

**Table 1:** General statistics for alignment and gene prediction

*Sample*	*Number of reads*	*Genes*	
		*With at least 4 × coverage*	*Avg. number of reads*
1	13 038 731	17 599	18.38
2	11 148 244	13 580	15.9
3	11 436 982	15 831	17.1
4	11 446 692	14 567	16.36
5	11 532 176	13 916	15.29
6	11 842 145	12 713	14.28
7	11 566 691	12 303	14.82
8	14 038 053	14 399	20.74
9	10 536 771	12 612	14.3
10	11 965 948	10 546	12.59
11	8 638 639	8 914	11.48
12	11 455 004	11 626	13.47
13	12 201 793	15 793	16.3
14	13 461 554	19 889	19.38
