# Divergent functional isoforms drive niche specialisation for nutrient acquisition and use in rumen microbiome

**DOI:** 10.1038/ismej.2017.34

**Published:** 2017-05-16

**Authors:** Francesco Rubino, Ciara Carberry, Sinéad M Waters, David Kenny, Matthew S McCabe, Christopher J Creevey

**Correction to:** *The ISME Journal* (2017) 11, 932–944; doi:10.1038/ismej.2016.172

**Updated online 28 February 2017:** This article was originally published under a CC BY-NC-SA 4.0 license, but has now been made available under a CC-BY 4.0 license. The PDF and HTML versions of the paper have been modified accordingly.

