# Supplementary figures and images for: Divergent functional isoforms drive niche specialisation for nutrient acquisition and use in rumen microbiome
Source: ISME J. 2017 Jan 13;11(4):932–44. doi: 10.1038/ismej.2016.172 (PMC5364355; doi:10.1038/ismej.2016.172)

Figure S1

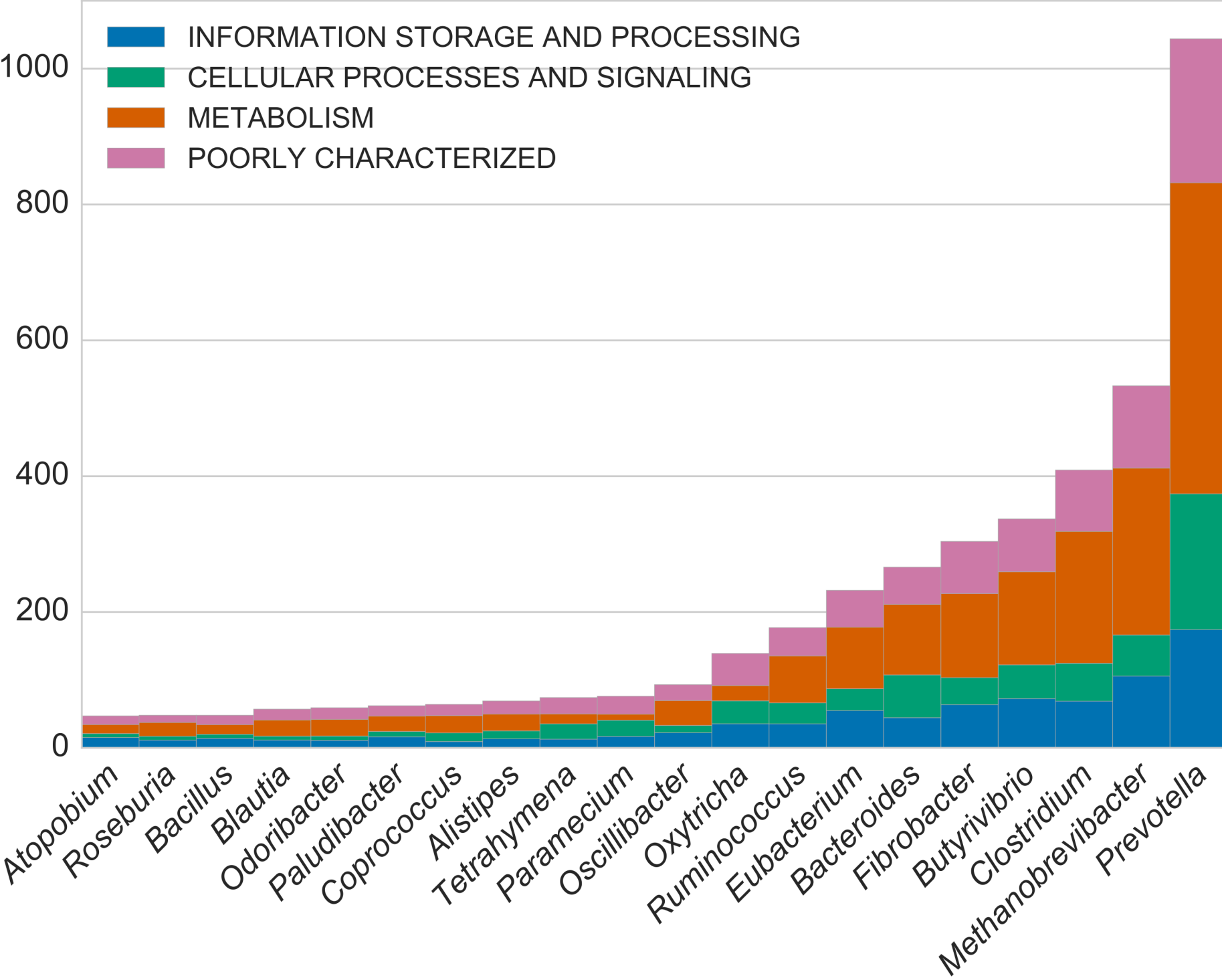

Supplement: Supplementary Figure S1 [file ismej2016172x1.pdf]

Figure S2

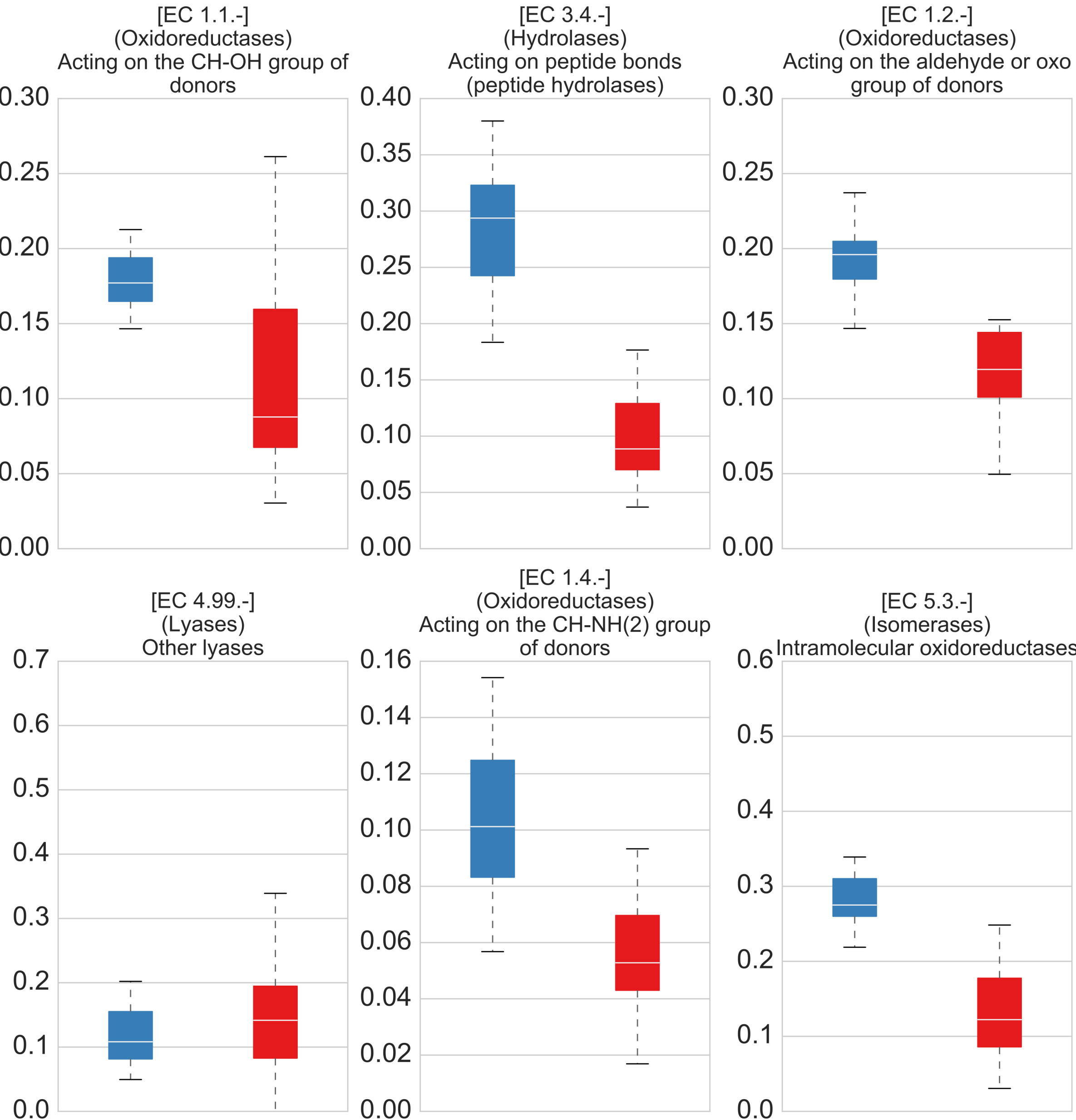

Supplement: Supplementary Figure S2 [file ismej2016172x2.pdf]

Figure S3

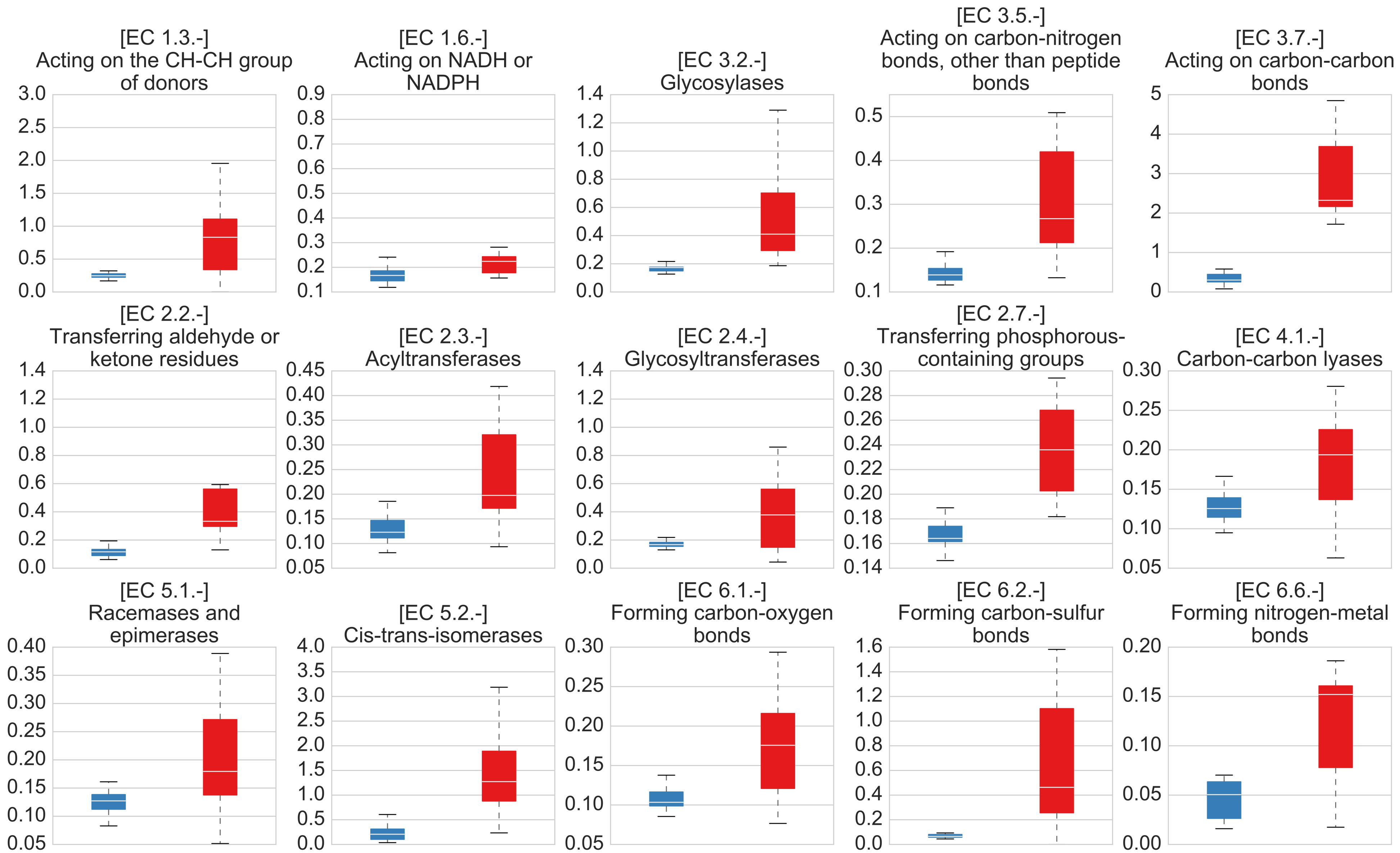

Supplement: Supplementary Figure S3 [file ismej2016172x3.pdf]
