# Supplementary material for: Divergent functional isoforms drive niche specialisation for nutrient acquisition and use in rumen microbiome
Source: ISME J. 2017 Jan 13;11(4):932–44. doi: 10.1038/ismej.2016.172 (PMC5364355; doi:10.1038/ismej.2016.172)
Supplement: Supplementary File 1 [file ismej2016172x10.html]

Simulation-bitscore


Used:
`diamond blastp -k 100 --sensitive --min-score 30 -q query.fa -d nr.dmnd -a output.daa`

Run on 10 subsamples of NCBI *NR*, and averaged the results.

The present notebook requires mgkit installed, preferably using the `pip install mgkit[full]`. The taxonomy is downloaded once by the `download-taxonomy.sh taxonomy.msgpack` included with it, in this case using the option of using *msgpack* to store it, for smaller size. Also seaborn should be installed.

In [1]:

```
from mgkit.io import blast
from mgkit import taxon
import mgkit
from glob import glob
import pandas as pd
import mgkit.plots
import seaborn as sns
```

In [2]:

```
mgkit.logger.config_log()
```

In [3]:

```
taxonomy = taxon.UniprotTaxonomy('taxonomy.msgpack')
```

```
2016-07-07 11:02:32,044 -    INFO - mgkit.taxon->load_data: Loading taxonomy from file taxonomy.msgpack
```

In [4]:

```
ncbi_ids = set()
```

In [5]:

```
split_header = lambda x: x.split('|')[3].split('.')[0]

sample_data = {}

for fname in glob('sample-000??.tab.gz'): # sample-0000?.tab.gz
    sample_name = fname.split('.')[0]
    
    data = []
    
    for query_id, (subject_id, bitscore) in blast.parse_blast_tab(fname, ret_col=[1, -1], key_func=split_header, value_funcs=[split_header, float]):
        ncbi_ids.add(query_id)
        data.append(
            (query_id, subject_id, bitscore)
        )
    
    sample_data[sample_name] = data
```

```
2016-07-07 11:02:36,042 -    INFO - mgkit.io.blast->parse_blast_tab: Reading BLAST results from file (sample-00001.tab.gz)
2016-07-07 11:02:44,133 -    INFO - mgkit.io.blast->parse_blast_tab: Read 1066660 BLAST records
2016-07-07 11:02:44,134 -    INFO - mgkit.io.blast->parse_blast_tab: Reading BLAST results from file (sample-00002.tab.gz)
2016-07-07 11:02:51,927 -    INFO - mgkit.io.blast->parse_blast_tab: Read 1050968 BLAST records
2016-07-07 11:02:51,928 -    INFO - mgkit.io.blast->parse_blast_tab: Reading BLAST results from file (sample-00003.tab.gz)
2016-07-07 11:02:59,944 -    INFO - mgkit.io.blast->parse_blast_tab: Read 1055403 BLAST records
2016-07-07 11:02:59,945 -    INFO - mgkit.io.blast->parse_blast_tab: Reading BLAST results from file (sample-00004.tab.gz)
2016-07-07 11:03:07,809 -    INFO - mgkit.io.blast->parse_blast_tab: Read 1048971 BLAST records
2016-07-07 11:03:07,809 -    INFO - mgkit.io.blast->parse_blast_tab: Reading BLAST results from file (sample-00005.tab.gz)
2016-07-07 11:03:15,558 -    INFO - mgkit.io.blast->parse_blast_tab: Read 1036303 BLAST records
2016-07-07 11:03:15,559 -    INFO - mgkit.io.blast->parse_blast_tab: Reading BLAST results from file (sample-00006.tab.gz)
2016-07-07 11:03:23,503 -    INFO - mgkit.io.blast->parse_blast_tab: Read 1055020 BLAST records
2016-07-07 11:03:23,504 -    INFO - mgkit.io.blast->parse_blast_tab: Reading BLAST results from file (sample-00007.tab.gz)
2016-07-07 11:03:31,417 -    INFO - mgkit.io.blast->parse_blast_tab: Read 1054779 BLAST records
2016-07-07 11:03:31,418 -    INFO - mgkit.io.blast->parse_blast_tab: Reading BLAST results from file (sample-00008.tab.gz)
2016-07-07 11:03:39,625 -    INFO - mgkit.io.blast->parse_blast_tab: Read 1060711 BLAST records
2016-07-07 11:03:39,626 -    INFO - mgkit.io.blast->parse_blast_tab: Reading BLAST results from file (sample-00009.tab.gz)
2016-07-07 11:03:47,407 -    INFO - mgkit.io.blast->parse_blast_tab: Read 1042581 BLAST records
2016-07-07 11:03:47,408 -    INFO - mgkit.io.blast->parse_blast_tab: Reading BLAST results from file (sample-00010.tab.gz)
2016-07-07 11:03:55,200 -    INFO - mgkit.io.blast->parse_blast_tab: Read 1054067 BLAST records
```

In [6]:

```
len(ncbi_ids)
```

Out[6]:

```
99888
```

In [7]:

```
taxon_ids = dict(blast.parse_accession_taxa_table('prot.accession2taxid.gz', acc_ids=ncbi_ids, key=0, num_lines=blast.NUM_LINES * 10))
```

```
2016-07-07 11:03:55,211 -    INFO - mgkit.io.blast->parse_accession_taxa_table: Reading taxonomic information from file (prot.accession2taxid.gz)
2016-07-07 11:04:20,701 -    INFO - mgkit.io.blast->parse_accession_taxa_table: Parsed 10000000 lines
2016-07-07 11:04:46,943 -    INFO - mgkit.io.blast->parse_accession_taxa_table: Parsed 20000000 lines
2016-07-07 11:05:12,834 -    INFO - mgkit.io.blast->parse_accession_taxa_table: Parsed 30000000 lines
2016-07-07 11:05:38,200 -    INFO - mgkit.io.blast->parse_accession_taxa_table: Parsed 40000000 lines
2016-07-07 11:06:03,545 -    INFO - mgkit.io.blast->parse_accession_taxa_table: Parsed 50000000 lines
2016-07-07 11:06:32,040 -    INFO - mgkit.io.blast->parse_accession_taxa_table: Parsed 60000000 lines
2016-07-07 11:06:57,973 -    INFO - mgkit.io.blast->parse_accession_taxa_table: Parsed 70000000 lines
2016-07-07 11:07:23,553 -    INFO - mgkit.io.blast->parse_accession_taxa_table: Parsed 80000000 lines
2016-07-07 11:07:49,301 -    INFO - mgkit.io.blast->parse_accession_taxa_table: Parsed 90000000 lines
2016-07-07 11:08:14,295 -    INFO - mgkit.io.blast->parse_accession_taxa_table: Parsed 100000000 lines
2016-07-07 11:08:40,817 -    INFO - mgkit.io.blast->parse_accession_taxa_table: Parsed 110000000 lines
2016-07-07 11:09:06,549 -    INFO - mgkit.io.blast->parse_accession_taxa_table: Parsed 120000000 lines
2016-07-07 11:09:31,936 -    INFO - mgkit.io.blast->parse_accession_taxa_table: Parsed 130000000 lines
2016-07-07 11:09:57,554 -    INFO - mgkit.io.blast->parse_accession_taxa_table: Parsed 140000000 lines
2016-07-07 11:10:23,439 -    INFO - mgkit.io.blast->parse_accession_taxa_table: Parsed 150000000 lines
2016-07-07 11:10:48,678 -    INFO - mgkit.io.blast->parse_accession_taxa_table: Parsed 160000000 lines
2016-07-07 11:11:14,438 -    INFO - mgkit.io.blast->parse_accession_taxa_table: Parsed 170000000 lines
2016-07-07 11:11:39,941 -    INFO - mgkit.io.blast->parse_accession_taxa_table: Parsed 180000000 lines
2016-07-07 11:12:05,120 -    INFO - mgkit.io.blast->parse_accession_taxa_table: Parsed 190000000 lines
2016-07-07 11:12:31,767 -    INFO - mgkit.io.blast->parse_accession_taxa_table: Parsed 200000000 lines
2016-07-07 11:12:58,110 -    INFO - mgkit.io.blast->parse_accession_taxa_table: Parsed 210000000 lines
2016-07-07 11:13:23,315 -    INFO - mgkit.io.blast->parse_accession_taxa_table: Parsed 220000000 lines
2016-07-07 11:13:48,157 -    INFO - mgkit.io.blast->parse_accession_taxa_table: Parsed 230000000 lines
2016-07-07 11:14:12,848 -    INFO - mgkit.io.blast->parse_accession_taxa_table: Parsed 240000000 lines
2016-07-07 11:14:37,472 -    INFO - mgkit.io.blast->parse_accession_taxa_table: Parsed 250000000 lines
2016-07-07 11:15:02,386 -    INFO - mgkit.io.blast->parse_accession_taxa_table: Parsed 260000000 lines
2016-07-07 11:15:28,211 -    INFO - mgkit.io.blast->parse_accession_taxa_table: Parsed 270000000 lines
2016-07-07 11:15:53,875 -    INFO - mgkit.io.blast->parse_accession_taxa_table: Parsed 280000000 lines
2016-07-07 11:16:19,746 -    INFO - mgkit.io.blast->parse_accession_taxa_table: Parsed 290000000 lines
```

In [8]:

```
def get_counts(data, taxonomy, rank='genus', min_bitscore=60):
    count_t = 0
    count_f = 0
    for query_id, subject_id, bitscore in data:
        if bitscore < min_bitscore:
            continue
        try:
            taxon_id1 = taxon_ids[query_id]
            taxon_id2 = taxon_ids[subject_id]
        except KeyError:
            continue
        if taxonomy.get_ranked_taxon(taxon_id1, rank) == taxonomy.get_ranked_taxon(taxon_id2, rank):
            count_t += 1
        else:
            count_f += 1

    return count_t, count_f
```

In [20]:

```
proportions = {}
for sample_name, data in sample_data.iteritems():
    sample_prop = {}
    for bitscore in xrange(30, 120, 5):
        count_t, count_f = get_counts(data, taxonomy, rank='genus', min_bitscore=bitscore)
        sample_prop[bitscore] = float(count_t) / (count_t + count_f)

    proportions[sample_name] = sample_prop
proportions = pd.DataFrame(proportions)
```

In [22]:

```
fig, ax = mgkit.plots.get_single_figure(figsize=(10, 5))
ax.plot(proportions.index, proportions.quantile(q=0.95, axis=1), color='g')
ax.plot(proportions.index, proportions.quantile(q=0.05, axis=1), color='g')
ax.fill_between(proportions.index, proportions.quantile(q=0.05, axis=1), proportions.quantile(q=0.95, axis=1), color='g', alpha=0.5)
ax.plot(proportions.index, proportions.median(axis=1), color='k')

# ax.boxplot(proportions.T.values, vert=True, positions=proportions.index, widths=1.5)
ax.set_xlim(left=25, right=120)
fig.savefig('plot-proportions.pdf')
```
