# Supplementary material for: Divergent functional isoforms drive niche specialisation for nutrient acquisition and use in rumen microbiome
Source: ISME J. 2017 Jan 13;11(4):932–44. doi: 10.1038/ismej.2016.172 (PMC5364355; doi:10.1038/ismej.2016.172)
Supplement: Supplementary File 2 [file ismej2016172x11.html]

Gene-Counts-Final


# Gene Counts¶

## Imports and Data Load¶

In [1]:

```
from __future__ import division
# Standard library imports
import cPickle as pickle
import functools
import textwrap
import json

# Other libraries imports
from numpy import log10
import numpy
import seaborn as sns
import pandas as pd
import requests

# MGKit imports
from mgkit.io import gff
import mgkit.simple_cache
from mgkit import snps, taxon, plots, kegg, logger
from mgkit.mappings import eggnog
import mgkit.snps.filter
import mgkit.snps.mapper
import mgkit.snps.funcs
import mgkit.counts.func
```

In [2]:

```
# Config log
logger.config_log()
```

In [3]:

```
# check if the version correspond
mgkit.check_version('0.2.2')
```

```
2016-07-26 17:17:30,070 - WARNING - mgkit->check_version: This was tested with MGKit version 0.2.2
```

In [4]:

```
# Data was save to disk before
kd = kegg.KeggData('data/kegg.pickle')
ko_names = kd.get_ko_names()  # Downloads names for KO (now deprecated, use get_id_names)

a = pickle.load(open('new_rfi_set.pickle','rb'))  # SNPs data
tx = taxon.UniprotTaxonomy('data/taxonomy_full.pickle')  # Taxonomy
eg = eggnog.Kegg2NogMapper('data/eggnog.pickle')  # Kegg to eggNOG mappings
eg_map = eg.get_ko_map()
rank = 'genus'  # The taxonomic rank to be used
min_num = 3  # minimum number of samples
labelfont = 12  # font size for figures
fig_multiplier = 2  # Multiplier (not deprecated)
```

```
2016-07-26 17:17:30,078 -    INFO - mgkit.kegg->load_data: Loading data from file data/kegg.pickle
2016-07-26 17:17:58,991 -    INFO - mgkit.taxon->load_data: Loading taxonomy from file data/taxonomy_full.pickle
2016-07-26 17:18:10,781 -    INFO - mgkit.mappings.eggnog->load_data: Loading data from data/eggnog.pickle
```

In [5]:

```
# Saves taxon_id for high level taxonomic units
bacteria_id = 2
archaea_id = tx.find_by_name('archaea')[0]
fungi_id = tx.find_by_name('fungi')[0]
protozoa_id = mgkit.taxon.PROTISTS.values() # Protozoa have more than one taxonmic "root"
```

```
2016-07-26 17:18:11,819 -   DEBUG - mgkit.taxon->gen_name_map: Generate name map
```

## Highest Mean Value¶

In [6]:

```
_ = mgkit.snps.conv_func.get_full_dataframe(a, tx, min_num=3, index_type='gene').mean(axis=1).sort(inplace=False)
print len(_[_ == 0]) / len(_) * 100
print _.index[-1], _[-1]
```

```
2016-07-26 17:18:14,066 -    INFO - mgkit.snps.funcs->combine_sample_snps: Analysing SNP from sample t1_b3
2016-07-26 17:18:14,592 -    INFO - mgkit.snps.funcs->combine_sample_snps: Analysing SNP from sample t1_b2
2016-07-26 17:18:15,175 -    INFO - mgkit.snps.funcs->combine_sample_snps: Analysing SNP from sample t1_b1
2016-07-26 17:18:15,777 -    INFO - mgkit.snps.funcs->combine_sample_snps: Analysing SNP from sample t1_b7
2016-07-26 17:18:16,277 -    INFO - mgkit.snps.funcs->combine_sample_snps: Analysing SNP from sample t1_b6
2016-07-26 17:18:16,793 -    INFO - mgkit.snps.funcs->combine_sample_snps: Analysing SNP from sample t1_b5
2016-07-26 17:18:17,422 -    INFO - mgkit.snps.funcs->combine_sample_snps: Analysing SNP from sample t1_b4
2016-07-26 17:18:18,143 -    INFO - mgkit.snps.funcs->combine_sample_snps: Analysing SNP from sample t4_b1
2016-07-26 17:18:18,769 -    INFO - mgkit.snps.funcs->combine_sample_snps: Analysing SNP from sample t4_b2
2016-07-26 17:18:19,238 -    INFO - mgkit.snps.funcs->combine_sample_snps: Analysing SNP from sample t4_b3
2016-07-26 17:18:19,678 -    INFO - mgkit.snps.funcs->combine_sample_snps: Analysing SNP from sample t4_b4
2016-07-26 17:18:20,064 -    INFO - mgkit.snps.funcs->combine_sample_snps: Analysing SNP from sample t4_b5
2016-07-26 17:18:20,507 -    INFO - mgkit.snps.funcs->combine_sample_snps: Analysing SNP from sample t4_b6
2016-07-26 17:18:21,080 -    INFO - mgkit.snps.funcs->combine_sample_snps: Analysing SNP from sample t4_b7
```

```
3.37674867342
K12586 7.30855855856
```

```
/Users/frubino/Dev/mgkit/dev-env/lib/python2.7/site-packages/ipykernel/__main__.py:1: FutureWarning: sort is deprecated, use sort_values(inplace=True) for INPLACE sorting
  if __name__ == '__main__':
```

## Isoforms count¶

In [7]:

```
# Loads all annotations
annotations = {x.uid: x for x in gff.parse_gff('rfi_samples-overlap-plen0_4-syn-cov-gc-scov-blast-NEW-CORR.gff')}
```

```
2016-07-26 17:18:38,671 -    INFO - mgkit.io.gff->parse_gff: Loading GFF from file (rfi_samples-overlap-plen0_4-syn-cov-gc-scov-blast-NEW-CORR.gff)
2016-07-26 17:18:47,553 -    INFO - mgkit.io.gff->parse_gff: Read 106475 line from file (rfi_samples-overlap-plen0_4-syn-cov-gc-scov-blast-NEW-CORR.gff)
```

In [8]:

```
# makes a list of ancestor taxon_id
anc_ids = [bacteria_id, archaea_id, fungi_id] + protozoa_id

count = {}
count_4x_cov = {}

for annotation in annotations.itervalues():
    # Returns the ranked taxon_id
    taxon_id = mgkit.counts.func.map_taxon_id_to_rank(tx, rank, annotation.taxon_id, include_higher=False)
    # if taxon_id is None, it means that the taxon_id was not resolved at the required level,
    # so we can exclude it from analysis
    if taxon_id is None:
        continue  
    
    # The counts are added per taxon_id to the two dictionaries, count
    # and the count_4x_cov for genes with a coverage of 4x at least
    for anc_id in anc_ids:
        if tx.is_ancestor(taxon_id, anc_id):
            if pd.Series(annotation.sample_coverage).mean() >= 4:
                try:
                    count_4x_cov[taxon_id].append(annotation.uid)
                except KeyError:
                    count_4x_cov[taxon_id] = [annotation.uid]
            try:
                count[taxon_id].append(annotation.uid)
            except KeyError:
                count[taxon_id] = [annotation.uid]
            break
```

In [9]:

```
len (count), len(set(count) & set(count_4x_cov))
```

Out[9]:

```
(826, 383)
```

In [10]:

```
len(annotations)
```

Out[10]:

```
106475
```

In [11]:

```
# Number of genera per each group
for anc_id, name in zip([bacteria_id, archaea_id, fungi_id, protozoa_id], ['Bacteria', 'Archaea', 'Fungi', 'Protozoa']):
    print name, '>=1 gene, 4x cov', sum(1 for taxon_id in count_4x_cov if tx.is_ancestor(taxon_id, anc_id))
```

```
Bacteria >=1 gene, 4x cov 261
Archaea >=1 gene, 4x cov 40
Fungi >=1 gene, 4x cov 47
Protozoa >=1 gene, 4x cov 35
```

# pN/pS (Genera)¶

In [12]:

```
# Defines custom filters to be used for combine_sample_snps
taxon_func = functools.partial(mgkit.snps.mapper.map_taxon_id_to_rank, taxonomy=tx, rank=rank)
filters = mgkit.snps.filter.get_default_filters(tx)
filters[-1] = functools.partial(
        mgkit.snps.filter.filter_genesyn_by_taxon_id,
        taxonomy=tx,
        filter_list=[
            mgkit.taxon.BACTERIA, 
            mgkit.taxon.ARCHAEA, 
            mgkit.taxon.FUNGI
        ] + mgkit.taxon.PROTISTS.values(),
        exclude=False,
        func=mgkit.taxon.is_ancestor
    )
# Builds a full dataframe, with index type (gene_id, taxon_id)
df = mgkit.snps.funcs.combine_sample_snps(a, min_num, filters, taxon_func=taxon_func, index_type=None)

########## check
taxon_ids = set(df.index.get_level_values('taxon'))
taxon_ids = sorted(taxon_ids)

dft = df.reorder_levels(['taxon', 'gene']).sortlevel(0)
txtot = {taxon_id: set(dft.loc[taxon_id].mean(axis=1).index) for taxon_id in taxon_ids}
porder = sorted(txtot, key=lambda x: len(txtot[x]))
```

```
2016-07-26 17:19:05,350 -    INFO - mgkit.snps.funcs->combine_sample_snps: Analysing SNP from sample t1_b3
2016-07-26 17:19:06,084 -    INFO - mgkit.snps.funcs->combine_sample_snps: Analysing SNP from sample t1_b2
2016-07-26 17:19:06,930 -    INFO - mgkit.snps.funcs->combine_sample_snps: Analysing SNP from sample t1_b1
2016-07-26 17:19:07,923 -    INFO - mgkit.snps.funcs->combine_sample_snps: Analysing SNP from sample t1_b7
2016-07-26 17:19:08,678 -    INFO - mgkit.snps.funcs->combine_sample_snps: Analysing SNP from sample t1_b6
2016-07-26 17:19:09,404 -    INFO - mgkit.snps.funcs->combine_sample_snps: Analysing SNP from sample t1_b5
2016-07-26 17:19:10,319 -    INFO - mgkit.snps.funcs->combine_sample_snps: Analysing SNP from sample t1_b4
2016-07-26 17:19:11,354 -    INFO - mgkit.snps.funcs->combine_sample_snps: Analysing SNP from sample t4_b1
2016-07-26 17:19:12,323 -    INFO - mgkit.snps.funcs->combine_sample_snps: Analysing SNP from sample t4_b2
2016-07-26 17:19:13,066 -    INFO - mgkit.snps.funcs->combine_sample_snps: Analysing SNP from sample t4_b3
2016-07-26 17:19:13,728 -    INFO - mgkit.snps.funcs->combine_sample_snps: Analysing SNP from sample t4_b4
2016-07-26 17:19:14,261 -    INFO - mgkit.snps.funcs->combine_sample_snps: Analysing SNP from sample t4_b5
2016-07-26 17:19:14,903 -    INFO - mgkit.snps.funcs->combine_sample_snps: Analysing SNP from sample t4_b6
2016-07-26 17:19:15,769 -    INFO - mgkit.snps.funcs->combine_sample_snps: Analysing SNP from sample t4_b7
```

In [13]:

```
# Calculate the number of genes
ngen = dft.mean(axis=1).count(level=0)
ngen = ngen[ngen > 3]
print "Bacteria genera:", sum(1 for taxon_id in ngen.index if tx.is_ancestor(taxon_id, 2))
print "Archaea genera:", sum(1 for taxon_id in ngen.index if tx.is_ancestor(taxon_id, tx.find_by_name('archaea')[0]))
print "Fungi genera:", sum(1 for taxon_id in ngen.index if tx.is_ancestor(taxon_id, tx.find_by_name('fungi')[0]))
print "Protozoa genera:", sum(1 for taxon_id in ngen.index if tx.is_ancestor(taxon_id, mgkit.taxon.PROTISTS.values()))
```

```
Bacteria genera: 127
Archaea genera: 20
Fungi genera: 5
Protozoa genera: 21
```

In [14]:

```
def get_ancestry(is_ancestor, taxon_id):
    for anc_id, label in zip((taxon.ARCHAEA, taxon.BACTERIA, taxon.FUNGI, tuple(taxon.PROTISTS.values())), ['Archaea', 'Bacteria', 'Fungi', 'Protozoa']):
        if is_ancestor(taxon_id, anc_id):
            return label
```

In [15]:

```
# Keep only genera with number of genes > 3
dft = dft.loc[list(ngen.index)].sortlevel('taxon')
```

## Table for Number of Genes per Genera¶

In [16]:

```
_ = (ngen / ngen.sum()*100)
print "Number of genes in Prevotella (%)", _.loc[838]
print "Number of genes in Methanobrevibacter (%)", _.loc[2172]
print "Number of genes in Clostridium (%)",  _.loc[1485]
```

```
Number of genes in Prevotella (%) 17.6619861276
Number of genes in Methanobrevibacter (%) 9.01708678735
Number of genes in Clostridium (%) 6.91930299442
```

In [17]:

```
cached_func = mgkit.simple_cache.memoize(tx.is_ancestor)
ngen_min1 = dft.mean(axis=1).count(level=0)
ngen_min1 = ngen_min1[ngen_min1 > 0]
gene_table = pd.DataFrame({
    ('Number of Genes', 'n'): ngen_min1,
    ('Number of Genes', '%'): ngen_min1 / ngen_min1.sum() * 100,
    ('Number of Isoforms', 'n'): {
        taxon_id: sum(1 for annotation in annotations.itervalues() if cached_func(annotation.taxon_id, taxon_id))
        for taxon_id in ngen_min1.index
    },
    ('Genomes', 'Lineage'): {
        taxon_id: get_ancestry(cached_func, taxon_id)
        for taxon_id in ngen_min1.index
    }
}).sort(('Number of Genes', 'n'), ascending=False)
```

```
/Users/frubino/Dev/mgkit/dev-env/lib/python2.7/site-packages/ipykernel/__main__.py:15: FutureWarning: sort(columns=....) is deprecated, use sort_values(by=.....)
```

In [18]:

```
gene_table[('Number of Isoforms', '%')] = gene_table[('Number of Isoforms', 'n')] / gene_table[('Number of Isoforms', 'n')].sum() * 100
```

### Get Average Genome Size from ENSEMBLE¶

In [19]:

```
# ensembl URL
url = "http://rest.ensemblgenomes.org/info/genomes/taxonomy/{}?content-type=application/json"
```

In [20]:

```
try:
    genome_info = pickle.load(open("genome-info.pickle", 'r'))
# useful when the notebook is first run (connection fails midway), but to reproduce
# the results at the time of writing it's best to leave have the saved copy
#     if len(genome_info) != len(ngen_min1.index):
#         raise IOError
except IOError:
    genome_info = {}
    # Downloads for each genus information about the
    # genomes available
    for idx, taxon_id in enumerate(ngen_min1.index):
        print idx + 1, len(ngen_min1), tx[taxon_id].s_name
        if taxon_id in genome_info:
            continue
        r = requests.get(url.format(taxon_id))
        if not r.ok:
            print r.status_code 
            continue
        genome_info[taxon_id] = r.json()
    pickle.dump(genome_info, open("genome-info.pickle", 'w'))
```

In [21]:

```
# Adds average genome size to table
gene_table[('Genomes', 'Average Size')] = pd.Series({
    taxon_id: pd.Series(int(genome['base_count']) for genome in genome_info[taxon_id]).mean()
    for taxon_id in ngen_min1.index
    if taxon_id in genome_info
})
```

In [22]:

```
# Adds two more columns using information in the dataframe
gene_table[('Number of Isoforms', 'Avg. Copies')] = gene_table[('Number of Isoforms', 'n')] / gene_table[('Number of Genes', 'n')]
gene_table[('Number of Genes', 'by Genome Size')] = gene_table[('Number of Genes', 'n')] / (gene_table[('Genomes', 'Average Size')] / gene_table[('Genomes', 'Average Size')].min())
```

In [23]:

```
gene_table.sort(axis=1, inplace=True)
```

```
/Users/frubino/Dev/mgkit/dev-env/lib/python2.7/site-packages/ipykernel/__main__.py:1: FutureWarning: sort(....) is deprecated, use sort_index(.....)
  if __name__ == '__main__':
```

In [24]:

```
print "Methanobrevibacter average genome size:", pd.Series(int(genome['base_count']) for genome in genome_info[2172]).mean()
print "Clostridium average genome size:", pd.Series(int(genome['base_count']) for genome in genome_info[1485]).mean()
print "Prevotella average genome size:", pd.Series(int(genome['base_count']) for genome in genome_info[838]).mean()
```

```
Methanobrevibacter average genome size: 2046215.0
Clostridium average genome size: 3904469.53191
Prevotella average genome size: 3024000.75
```

In [25]:

```
# Reorder columns
gene_table = gene_table[['Number of Genes', 'Number of Isoforms', 'Genomes']]
```

In [26]:

```
gene_table.index.name = 'Genus'
```

In [27]:

```
# Makes Latex table
gene_table.rename(index={taxon_id: tx[taxon_id].s_name.capitalize() for taxon_id in ngen_min1.index}).to_csv('number_of_genes.csv')

# Probably not all of them
gene_table.rename(
    index={taxon_id: "\emph{{{}}}".format(tx[taxon_id].s_name.capitalize()) for taxon_id in ngen_min1.index},
    columns=lambda x: x.replace('%', r'\%')
).iloc[:25].to_latex(
    'number_of_genes.tex', 
    longtable=False, 
    float_format=lambda x: 'NA' if pd.isnull(x) else "{:.2f}".format(x), 
    na_rep="NA",
    escape=False, # to use emphasis in the index names
)
```

In [28]:

```
_ = ngen.loc[[taxon_id for taxon_id in ngen.index if tx.is_ancestor(taxon_id, mgkit.taxon.ARCHAEA)]].sum()
print "Percentage of Methanobrevibacter genes of all Archaea genes", ngen.loc[2172] / _ * 100
```

```
Percentage of Methanobrevibacter genes of all Archaea genes 76.4705882353
```

## Table for all pN/pS Values¶

In [29]:

```
dft.rename(
    index=lambda x: tx[x].s_name.capitalize() if x in tx else x
).sortlevel(0, inplace=False).to_csv('pnps_all.csv')
```

## Table for pN/pS Mean Values, with Variance¶

In [30]:

```
mean_pnps = pd.DataFrame({
    ('pN/pS', 'Mean'): dft.mean(axis=1),
    ('pN/pS', 'Std'): dft.std(axis=1),
    ('pN/pS', 'Min'): dft.min(axis=1),    
    ('pN/pS', 'Max'): dft.max(axis=1),    
    ('Number of Samples', ''): dft.count(axis=1),
}).rename(index=lambda x: tx[x].s_name.capitalize() if x in tx else x)
mean_pnps = mean_pnps[
    [('Number of Samples', ''), ('pN/pS', 'Mean'), ('pN/pS', 'Std'), ('pN/pS', 'Min'), ('pN/pS', 'Max')]
].sortlevel(0)
mean_pnps.index.names = ['Taxon', 'Gene Id']
```

In [31]:

```
mean_pnps.to_csv('mean_pnps.csv')
```

# eggNOG Counts¶

In [58]:

```
gene_cat
```

Out[58]:

```
{1, 2}
```

In [32]:

```
# tx_eggnog = {}

# for taxon_id in taxon_ids:
#     # don't include any genera that is not a leaf of one of the accepted groups
#     if not tx.is_ancestor(taxon_id, [bacteria_id, archaea_id, fungi_id] + protozoa_id):
#         continue
        
#     tx_eggnog[taxon_id] = {x: 0 for x in range(len(eggnog.EGGNOG_CAT_KEYS))}

#     for gene_id in dft.loc[taxon_id].index:
#         gene_cat = set()
#         try:
#             for category in eg_map[gene_id]:
#                 for idx, categories in enumerate(eggnog.EGGNOG_CAT_KEYS):
#                     if category in categories:
#                         gene_cat.add(idx)
#                         break
#         except KeyError:
#             # a missing gene in the mappings counts as a 'poorly characterized'
#             gene_cat.add(3)
#         # print gene_id, gene_cat
#         for category in gene_cat:
#             tx_eggnog[taxon_id][category] += 1
```

In [63]:

```
tx_eggnog = {}

for taxon_id in taxon_ids:
    # don't include any genera that is not a leaf of one of the accepted groups
    if not tx.is_ancestor(taxon_id, [bacteria_id, archaea_id, fungi_id] + protozoa_id):
        continue
        
    tx_eggnog[taxon_id] = {x: 0 for x in range(len(eggnog.EGGNOG_CAT_KEYS))}

    for gene_id in dft.loc[taxon_id].index:
        gene_cat = set()
        try:
            for category in eg_map[gene_id]:
                for idx, categories in enumerate(eggnog.EGGNOG_CAT_KEYS):
                    if category in categories:
                        gene_cat.add(idx)
                        break
        except KeyError:
            # a missing gene in the mappings counts as a 'poorly characterized'
            gene_cat.add(3)
        # print gene_id, gene_cat
        for category in gene_cat:
            tx_eggnog[taxon_id][category] += 1. / len(gene_cat)
```

In [64]:

```
count_eg = {x: 0. for x in xrange(4)}
for d in tx_eggnog.itervalues():
    for key, value in d.iteritems():
        count_eg[key] += value
for index, name in enumerate(eggnog.EGGNOG_CAT_NAMES):
    print name, '(%)', count_eg[index] / sum(count_eg.values()) * 100
```

```
INFORMATION STORAGE AND PROCESSING (%) 20.2165454238
CELLULAR PROCESSES AND SIGNALING (%) 16.9401680483
METABOLISM (%) 38.8597530029
POORLY CHARACTERIZED (%) 23.9835335251
```

In [34]:

```
# Gene with highest pN/pS
taxon_id, gene_id = dft[dft.max(axis=1) == dft.max(axis=1).max()].index[0]
print tx[taxon_id].s_name, gene_id
```

```
prevotella K15633
```

In [35]:

```
print "Max pN/pS", dft.max().max()
print "Percentage of gene with a pN/pS = 0 in at least 1 gene", len(dft[dft.min(axis=1) == 0]) / len(dft) * 100
```

```
Max pN/pS 27.1621621622
Percentage of gene with a pN/pS = 0 in at least 1 gene 59.8037557097
```

In [66]:

```
sns.set_style('whitegrid')

# Saves the dictionary to disk
f = open('eggnog_genes.txt', 'w')
f.write('taxon\t{}\Lineage\tTotal genes\n'.format('\t'.join(eggnog.EGGNOG_CAT_NAMES)))
for taxon_id, cat_counts in tx_eggnog.iteritems():
    
    f.write('{}\t{}\t{}\t{}\n'.format(
        tx[taxon_id].s_name,
        '\t'.join(str(cat_counts[idx]) for idx in range(4)),
        ':'.join(tx[taxon_id].lineage),
        len(txtot[taxon_id])
    )
    )

f.close()

fig, ax = mgkit.plots.get_single_figure(dpi=300, figsize=(10, 8))

porder = sorted(txtot, key=lambda x: len(txtot[x]))
porder = porder[-20:]
print len(porder)

values = numpy.zeros(len(porder))
totals = numpy.array([sum(tx_eggnog[taxon_id].values()) for taxon_id in porder], dtype=numpy.float)
width = 1.0

# colors = ['#E41A1C', '#377EB8', '#4DAF4A', '#984EA3']
colors = sns.color_palette('colorblind', 4)

for idx, color in enumerate(colors):
    cat_values = numpy.array([tx_eggnog[taxon_id][idx] for taxon_id in porder])
    #to use percentages uncomment
    # cat_values = cat_values / totals
    ax.bar(
        range(len(porder)),
        cat_values,
        width=width,
        color=color,
        bottom=values,
        label=eggnog.EGGNOG_CAT_NAMES[idx],
        edgecolor='#AAAAAA'
    )
    values += cat_values

for text in ax.get_yticklabels():
    text.set_fontsize(18)
    
ax.set_xticks(numpy.arange(len(porder)) + (width / 2.0) + .25)
ax.set_xticklabels([tx[taxon_id].s_name.capitalize() for taxon_id in porder],
                   rotation=45, fontsize=18, style='italic', ha='right')

ax.grid(which='major', axis='x')
ax.legend(loc='upper left', fontsize=16)
ax.set_ylim(top=1100)
fig.tight_layout()
fig.savefig('eggnog_genes.pdf', bbox_inches='tight')
```

```
20
```

## Comparison of Number of Genes - Raw vs Scaled by Genome Size (average)¶

In [37]:

```
col1 = ('Number of Genes', 'n')
col2 = ('Number of Genes', 'by Genome Size')

_ = gene_table[col1].sort(ascending=False, inplace=False).index
_ = gene_table[col2].loc[_].dropna().index#[:12]

color1 = [sns.saturate(x) for x in sns.color_palette('hls', 12)]
color2 = sns.color_palette('hls', 12)

tmp = gene_table[col1].loc[_]
tmp2 = gene_table[col2].loc[_]
tmp = tmp / tmp.sum()
tmp2 = tmp2 / tmp2.sum()

tmp = tmp.iloc[:10]
tmp2 = tmp2.iloc[:10]

fig, ax = mgkit.plots.get_single_figure(figsize=(20, 10))

ax.bar(range(len(tmp)), tmp, width=.4, color=color1, lw=1)
ax.bar(numpy.arange(len(tmp))+0.4, tmp2, width=0.4, color=color2, lw=1)
_ = ax.set_xticks(numpy.arange(0.4, len(tmp)))
_ = ax.set_xticklabels(
    [
        tx[taxon_id].s_name.capitalize() 
        for taxon_id in tmp.index
    ], 
    rotation=35,
    ha='right'
)
_ = ax.set_yticklabels(
    ["{}%".format(x * 100) for x in ax.get_yticks()]
)

for text in ax.get_xticklabels() + ax.get_yticklabels():
    text.set_fontsize(22)
# text.set_style('italic')
ax.grid(axis='x')
ax.set_xlabel("Genera", fontsize=28)
ax.set_ylabel("Abundance", fontsize=28)
fig.tight_layout()
fig.savefig('Gene_numbers-with-scaled.pdf')
```

```
/Users/frubino/Dev/mgkit/dev-env/lib/python2.7/site-packages/ipykernel/__main__.py:4: FutureWarning: sort is deprecated, use sort_values(inplace=True) for INPLACE sorting
```

# Core Rumen Metagenome¶

In [38]:

```
dft_min14 = dft.dropna()
```

In [39]:

```
print "Number of genes:", len(dft_min14)
```

```
Number of genes: 519
```

In [40]:

```
ngen_min14 = dft_min14.mean(axis=1).sortlevel('taxon').groupby(level='taxon').count()
```

In [41]:

```
print "Number of genera", len(ngen_min14)
print "Number of archaeal genera", sum(1 for x in ngen_min14.index if tx.is_ancestor(x, mgkit.taxon.ARCHAEA))
print "Number of bacterial genera", sum(1 for x in ngen_min14.index if tx.is_ancestor(x, mgkit.taxon.BACTERIA))
```

```
Number of genera 49
Number of archaeal genera 6
Number of bacterial genera 43
```

In [42]:

```
ngen_min14.sort(ascending=False, inplace=True)
```

```
/Users/frubino/Dev/mgkit/dev-env/lib/python2.7/site-packages/ipykernel/__main__.py:1: FutureWarning: sort is deprecated, use sort_values(inplace=True) for INPLACE sorting
  if __name__ == '__main__':
```

In [43]:

```
top_genera = list(ngen_min14.index[:9])
print "Percentage of genes in the core from the top12 genera", len(dft_min14.loc[top_genera]) / len(dft_min14)
print "Number of archael genera in the top12", sum(1 for x in top_genera if tx.is_ancestor(x, mgkit.taxon.ARCHAEA))
print "Number of bacterial genera in the top12", sum(1 for x in top_genera if tx.is_ancestor(x, mgkit.taxon.BACTERIA))
```

```
Percentage of genes in the core from the top12 genera 0.899807321773
Number of archael genera in the top12 1
Number of bacterial genera in the top12 8
```

In [44]:

```
tx_eggnog14 = {taxon_id: {eid: 0 for eid in eggnog.EGGNOG_CAT_MAP} for taxon_id in ngen_min14.index}
ko2fc = mgkit.utils.dictionary.combine_dict(eg_map, mgkit.utils.dictionary.reverse_mapping(eggnog.EGGNOG_CAT_MAP))
for taxon_id in tx_eggnog14:
    for gene_id in dft_min14.loc[taxon_id].index:
        if gene_id not in ko2fc:
            continue
        for fcid in ko2fc[gene_id]:
            tx_eggnog14[taxon_id][fcid] += 1
```

In [45]:

```
min14table = pd.DataFrame(
{
    ('Genes', 'Percentage of Total (%)'): {
        taxon_id: ngen_min14.loc[taxon_id] / ngen_min14.sum() * 100
        for taxon_id in ngen_min14.index
    },
    ('Genes', 'Number'): {
        taxon_id: ngen_min14.loc[taxon_id]
        for taxon_id in ngen_min14.index
    },
    ('eggNOG', 'INFORMATION STORAGE AND PROCESSING'.title()): {
        taxon_id: tx_eggnog14[taxon_id]['INFORMATION STORAGE AND PROCESSING']
        for taxon_id in ngen_min14.index
    },
    ('eggNOG', 'CELLULAR PROCESSES AND SIGNALING'.title()): {
        taxon_id: tx_eggnog14[taxon_id]['CELLULAR PROCESSES AND SIGNALING']
        for taxon_id in ngen_min14.index
    },
    ('eggNOG', 'METABOLISM'.title()): {
        taxon_id: tx_eggnog14[taxon_id]['METABOLISM']
        for taxon_id in ngen_min14.index
    },
    ('eggNOG', 'POORLY CHARACTERIZED'.title()): {
        taxon_id: tx_eggnog14[taxon_id]['POORLY CHARACTERIZED']
        for taxon_id in ngen_min14.index
    },
}
)
```

In [46]:

```
min14table = min14table.icol([1, 0, 3, 2, -2, -1]).rename(index={x: tx[x].s_name.capitalize() for x in min14table.index})
min14table.sort(columns=[('Genes', 'Percentage of Total (%)')], ascending=False, inplace=True)
```

```
/Users/frubino/Dev/mgkit/dev-env/lib/python2.7/site-packages/ipykernel/__main__.py:1: FutureWarning: icol(i) is deprecated. Please use .iloc[:,i]
  if __name__ == '__main__':
/Users/frubino/Dev/mgkit/dev-env/lib/python2.7/site-packages/ipykernel/__main__.py:2: FutureWarning: sort(columns=....) is deprecated, use sort_values(by=.....)
  from ipykernel import kernelapp as app
```

In [47]:

```
min14table.rename(
    index=lambda x: "\emph{%s}" % x,
    columns=lambda x: x.replace('%', '\%')
).to_latex(
    'core-community.tex', 
    float_format=lambda x: "{:.2f}".format(x),
    escape=False
)
min14table.to_csv('core-community.csv')
```

In [48]:

```
min14table['eggNOG'].div(min14table['eggNOG'].sum(axis=1), axis='index').mean(axis='index') * 100
```

Out[48]:

```
Information Storage And Processing    25.754507
Cellular Processes And Signaling      14.229586
Metabolism                            35.435321
Poorly Characterized                  24.580586
dtype: float64
```
