# Supplementary material for: Divergent functional isoforms drive niche specialisation for nutrient acquisition and use in rumen microbiome
Source: ISME J. 2017 Jan 13;11(4):932–44. doi: 10.1038/ismej.2016.172 (PMC5364355; doi:10.1038/ismej.2016.172)
Supplement: Supplementary File 3 [file ismej2016172x12.html]

Gene-Variation-Paper


In [19]:

```
from __future__ import division
import cPickle as pickle
import textwrap

import seaborn as sns
import matplotlib.pyplot as plt
import pandas as pd
import numpy as np

import mgkit
from mgkit.snps.funcs import order_ratios
import mgkit.snps
from mgkit.mappings import eggnog
from mgkit.snps.conv_func import get_rank_dataframe, get_full_dataframe, get_gene_map_dataframe
from mgkit.utils import dictionary
import mgkit.plots
```

In [20]:

```
mgkit.check_version('0.2.2')
```

In [2]:

```
mgkit.logger.config_log()
```

In [3]:

```
tx = mgkit.taxon.UniprotTaxonomy('data/taxonomy_full.pickle')
```

```
INFO:mgkit.taxon:Loading taxonomy from file data/taxonomy_full.pickle
2016-01-26 11:06:32,250 -    INFO - mgkit.taxon->load_data: Loading taxonomy from file data/taxonomy_full.pickle
```

In [4]:

```
snp_data = pickle.load(open('new_rfi_set.pickle', 'rb'))
```

In [5]:

```
df = get_full_dataframe(snp_data, tx, index_type=None)
```

```
INFO:mgkit.snps.funcs:Analysing SNP from sample t1_b3
2016-01-26 11:07:12,326 -    INFO - mgkit.snps.funcs->combine_sample_snps: Analysing SNP from sample t1_b3
INFO:mgkit.snps.funcs:Analysing SNP from sample t1_b2
2016-01-26 11:07:13,432 -    INFO - mgkit.snps.funcs->combine_sample_snps: Analysing SNP from sample t1_b2
INFO:mgkit.snps.funcs:Analysing SNP from sample t1_b1
2016-01-26 11:07:14,707 -    INFO - mgkit.snps.funcs->combine_sample_snps: Analysing SNP from sample t1_b1
INFO:mgkit.snps.funcs:Analysing SNP from sample t1_b7
2016-01-26 11:07:16,289 -    INFO - mgkit.snps.funcs->combine_sample_snps: Analysing SNP from sample t1_b7
INFO:mgkit.snps.funcs:Analysing SNP from sample t1_b6
2016-01-26 11:07:17,439 -    INFO - mgkit.snps.funcs->combine_sample_snps: Analysing SNP from sample t1_b6
INFO:mgkit.snps.funcs:Analysing SNP from sample t1_b5
2016-01-26 11:07:18,537 -    INFO - mgkit.snps.funcs->combine_sample_snps: Analysing SNP from sample t1_b5
INFO:mgkit.snps.funcs:Analysing SNP from sample t1_b4
2016-01-26 11:07:19,868 -    INFO - mgkit.snps.funcs->combine_sample_snps: Analysing SNP from sample t1_b4
INFO:mgkit.snps.funcs:Analysing SNP from sample t4_b1
2016-01-26 11:07:21,280 -    INFO - mgkit.snps.funcs->combine_sample_snps: Analysing SNP from sample t4_b1
INFO:mgkit.snps.funcs:Analysing SNP from sample t4_b2
2016-01-26 11:07:22,639 -    INFO - mgkit.snps.funcs->combine_sample_snps: Analysing SNP from sample t4_b2
INFO:mgkit.snps.funcs:Analysing SNP from sample t4_b3
2016-01-26 11:07:23,708 -    INFO - mgkit.snps.funcs->combine_sample_snps: Analysing SNP from sample t4_b3
INFO:mgkit.snps.funcs:Analysing SNP from sample t4_b4
2016-01-26 11:07:24,727 -    INFO - mgkit.snps.funcs->combine_sample_snps: Analysing SNP from sample t4_b4
INFO:mgkit.snps.funcs:Analysing SNP from sample t4_b5
2016-01-26 11:07:25,539 -    INFO - mgkit.snps.funcs->combine_sample_snps: Analysing SNP from sample t4_b5
INFO:mgkit.snps.funcs:Analysing SNP from sample t4_b6
2016-01-26 11:07:26,497 -    INFO - mgkit.snps.funcs->combine_sample_snps: Analysing SNP from sample t4_b6
INFO:mgkit.snps.funcs:Analysing SNP from sample t4_b7
2016-01-26 11:07:27,752 -    INFO - mgkit.snps.funcs->combine_sample_snps: Analysing SNP from sample t4_b7
```

In [6]:

```
df.max().max(), df.min().min()
```

Out[6]:

```
(27.587921847246889, 0.0)
```

# eggNOG Variation¶

In [7]:

```
eg = eggnog.Kegg2NogMapper('data/eggnog.pickle')
eg_map = eg.get_ko_map()
```

```
INFO:mgkit.mappings.eggnog:Loading data from data/eggnog.pickle
2016-01-26 11:07:48,129 -    INFO - mgkit.mappings.eggnog->load_data: Loading data from data/eggnog.pickle
```

In [8]:

```
egdf = get_gene_map_dataframe(snp_data, tx, eg_map)
```

```
INFO:mgkit.snps.funcs:Analysing SNP from sample t1_b3
2016-01-26 11:07:52,291 -    INFO - mgkit.snps.funcs->combine_sample_snps: Analysing SNP from sample t1_b3
INFO:mgkit.snps.funcs:Analysing SNP from sample t1_b2
2016-01-26 11:07:52,744 -    INFO - mgkit.snps.funcs->combine_sample_snps: Analysing SNP from sample t1_b2
INFO:mgkit.snps.funcs:Analysing SNP from sample t1_b1
2016-01-26 11:07:53,202 -    INFO - mgkit.snps.funcs->combine_sample_snps: Analysing SNP from sample t1_b1
INFO:mgkit.snps.funcs:Analysing SNP from sample t1_b7
2016-01-26 11:07:53,653 -    INFO - mgkit.snps.funcs->combine_sample_snps: Analysing SNP from sample t1_b7
INFO:mgkit.snps.funcs:Analysing SNP from sample t1_b6
2016-01-26 11:07:54,036 -    INFO - mgkit.snps.funcs->combine_sample_snps: Analysing SNP from sample t1_b6
INFO:mgkit.snps.funcs:Analysing SNP from sample t1_b5
2016-01-26 11:07:54,379 -    INFO - mgkit.snps.funcs->combine_sample_snps: Analysing SNP from sample t1_b5
INFO:mgkit.snps.funcs:Analysing SNP from sample t1_b4
2016-01-26 11:07:54,897 -    INFO - mgkit.snps.funcs->combine_sample_snps: Analysing SNP from sample t1_b4
INFO:mgkit.snps.funcs:Analysing SNP from sample t4_b1
2016-01-26 11:07:55,442 -    INFO - mgkit.snps.funcs->combine_sample_snps: Analysing SNP from sample t4_b1
INFO:mgkit.snps.funcs:Analysing SNP from sample t4_b2
2016-01-26 11:07:55,911 -    INFO - mgkit.snps.funcs->combine_sample_snps: Analysing SNP from sample t4_b2
INFO:mgkit.snps.funcs:Analysing SNP from sample t4_b3
2016-01-26 11:07:56,254 -    INFO - mgkit.snps.funcs->combine_sample_snps: Analysing SNP from sample t4_b3
INFO:mgkit.snps.funcs:Analysing SNP from sample t4_b4
2016-01-26 11:07:56,556 -    INFO - mgkit.snps.funcs->combine_sample_snps: Analysing SNP from sample t4_b4
INFO:mgkit.snps.funcs:Analysing SNP from sample t4_b5
2016-01-26 11:07:56,826 -    INFO - mgkit.snps.funcs->combine_sample_snps: Analysing SNP from sample t4_b5
INFO:mgkit.snps.funcs:Analysing SNP from sample t4_b6
2016-01-26 11:07:57,159 -    INFO - mgkit.snps.funcs->combine_sample_snps: Analysing SNP from sample t4_b6
INFO:mgkit.snps.funcs:Analysing SNP from sample t4_b7
2016-01-26 11:07:57,578 -    INFO - mgkit.snps.funcs->combine_sample_snps: Analysing SNP from sample t4_b7
```

In [9]:

```
eg_major = dictionary.combine_dict(eg_map, dictionary.reverse_mapping(eggnog.EGGNOG_CAT_MAP))
```

In [25]:

```
egdf = get_gene_map_dataframe(snp_data, tx, eg_major)
```

```
INFO:mgkit.snps.funcs:Analysing SNP from sample t1_b3
2016-01-26 11:12:59,796 -    INFO - mgkit.snps.funcs->combine_sample_snps: Analysing SNP from sample t1_b3
INFO:mgkit.snps.funcs:Analysing SNP from sample t1_b2
2016-01-26 11:13:00,239 -    INFO - mgkit.snps.funcs->combine_sample_snps: Analysing SNP from sample t1_b2
INFO:mgkit.snps.funcs:Analysing SNP from sample t1_b1
2016-01-26 11:13:00,664 -    INFO - mgkit.snps.funcs->combine_sample_snps: Analysing SNP from sample t1_b1
INFO:mgkit.snps.funcs:Analysing SNP from sample t1_b7
2016-01-26 11:13:01,102 -    INFO - mgkit.snps.funcs->combine_sample_snps: Analysing SNP from sample t1_b7
INFO:mgkit.snps.funcs:Analysing SNP from sample t1_b6
2016-01-26 11:13:01,472 -    INFO - mgkit.snps.funcs->combine_sample_snps: Analysing SNP from sample t1_b6
INFO:mgkit.snps.funcs:Analysing SNP from sample t1_b5
2016-01-26 11:13:01,810 -    INFO - mgkit.snps.funcs->combine_sample_snps: Analysing SNP from sample t1_b5
INFO:mgkit.snps.funcs:Analysing SNP from sample t1_b4
2016-01-26 11:13:02,336 -    INFO - mgkit.snps.funcs->combine_sample_snps: Analysing SNP from sample t1_b4
INFO:mgkit.snps.funcs:Analysing SNP from sample t4_b1
2016-01-26 11:13:02,895 -    INFO - mgkit.snps.funcs->combine_sample_snps: Analysing SNP from sample t4_b1
INFO:mgkit.snps.funcs:Analysing SNP from sample t4_b2
2016-01-26 11:13:03,331 -    INFO - mgkit.snps.funcs->combine_sample_snps: Analysing SNP from sample t4_b2
INFO:mgkit.snps.funcs:Analysing SNP from sample t4_b3
2016-01-26 11:13:03,657 -    INFO - mgkit.snps.funcs->combine_sample_snps: Analysing SNP from sample t4_b3
INFO:mgkit.snps.funcs:Analysing SNP from sample t4_b4
2016-01-26 11:13:03,951 -    INFO - mgkit.snps.funcs->combine_sample_snps: Analysing SNP from sample t4_b4
INFO:mgkit.snps.funcs:Analysing SNP from sample t4_b5
2016-01-26 11:13:04,209 -    INFO - mgkit.snps.funcs->combine_sample_snps: Analysing SNP from sample t4_b5
INFO:mgkit.snps.funcs:Analysing SNP from sample t4_b6
2016-01-26 11:13:04,530 -    INFO - mgkit.snps.funcs->combine_sample_snps: Analysing SNP from sample t4_b6
INFO:mgkit.snps.funcs:Analysing SNP from sample t4_b7
2016-01-26 11:13:04,928 -    INFO - mgkit.snps.funcs->combine_sample_snps: Analysing SNP from sample t4_b7
```

In [26]:

```
egdf.rename(
    index=lambda x: '\n'.join(textwrap.wrap(x.capitalize(), 20)), #{x: x.capitalize() for x in eggnog.EGGNOG_CAT_MAP}, 
    inplace=True
)
```

In [27]:

```
egdf.mean(axis=1)
```

Out[27]:

```
Poorly characterized                   0.191798
Information storage\nand processing    0.185704
Metabolism                             0.180848
Cellular processes\nand signaling      0.193628
dtype: float64
```

In [30]:

```
reload(mgkit.plots)
colors = ['#E41A1C', '#377EB8', '#4DAF4A', '#984EA3']
sns.set_style('whitegrid')
fig, ax = mgkit.plots.get_single_figure(figsize=(15, 7.5), dpi=300)
plot_data = mgkit.plots.boxplot_dataframe(
    egdf, 
    egdf.index, 
    ax, 
    fonts=dict(fontsize=32, rotation=0),
    data_colours=dict(zip(egdf.index, colors)),
    box_vert=False,
    fill_box=True
)
ax.grid(axis='y')
fig.tight_layout()
fig.savefig('eggnog-major-boxplot-v1.pdf', bbox_inches='tight')
```

In [14]:

```
colors = ['#E41A1C', '#377EB8', '#4DAF4A', '#984EA3']
sns.set_style('whitegrid')
fig, ax = mgkit.plots.get_single_figure(figsize=(20, 10), dpi=300)
plot_data = mgkit.plots.boxplot_dataframe(
    egdf, 
    egdf.index, 
    ax, 
    fonts=dict(fontsize=32, rotation=0),
    data_colours=dict(zip(egdf.index, colors)),
    box_vert=False,
    fill_box=False
)
ax.grid(axis='y')
mgkit.plots.add_values_to_boxplot(
    egdf, 
    ax, 
    plot_data, 
    egdf.index,
    data_colours=dict(zip(egdf.index, colors)),
    s=1000,
    box_vert=False
)
fig.tight_layout()
fig.savefig('eggnog-major-boxplot-v2.pdf', bbox_inches='tight')
```

## Gene Variation¶

In [15]:

```
gvar = df.swaplevel(0, 1).sortlevel('taxon')
```

In [16]:

```
gvar.index.names = ['taxon_id', 'gene_id']
```

In [17]:

```
gvar.to_excel('gene-variation.xlsx')
```
