# Supplementary material for: Divergent functional isoforms drive niche specialisation for nutrient acquisition and use in rumen microbiome
Source: ISME J. 2017 Jan 13;11(4):932–44. doi: 10.1038/ismej.2016.172 (PMC5364355; doi:10.1038/ismej.2016.172)
Supplement: Supplementary File 4 [file ismej2016172x13.html]

Venn-Sign\_genes-Final


# Venn Diagram and 3 Way Test¶

## Import and Data Load¶

In [11]:

```
from __future__ import division

# Standard library
import cPickle as pickle
import functools

# External Libraries
import pandas
import pyperclip
from numpy import log10
import pandas
from scipy.stats import ks_2samp, ttest_1samp, ranksums
import matplotlib
import matplotlib.pyplot as plt
from matplotlib.gridspec import GridSpec
from matplotlib_venn import venn3

# MGKit imports
import mgkit
from mgkit.utils import r_func
import mgkit.kegg
import mgkit.taxon
import mgkit.snps.funcs
import mgkit.snps.filter
import mgkit.snps.mapper
```

In [2]:

```
# Log config
mgkit.logger.config_log()
```

In [4]:

```
##data load
kd = mgkit.kegg.KeggData('data/kegg.pickle')
ko_names = kd.get_ko_names()
a = pickle.load(open('new_rfi_set.pickle', 'rb'))
tx = mgkit.taxon.UniprotTaxonomy('data/taxonomy_full.pickle')
```

```
INFO:mgkit.kegg:Loading data from file data/kegg.pickle
2016-01-26 12:18:22,284 -    INFO - mgkit.kegg->load_data: Loading data from file data/kegg.pickle
INFO:mgkit.taxon:Loading taxonomy from file data/taxonomy_full.pickle
2016-01-26 12:18:55,469 -    INFO - mgkit.taxon->load_data: Loading taxonomy from file data/taxonomy_full.pickle
```

## Build Table¶

In [5]:

```
###### dataframe build
min_num = 3

taxon_func = functools.partial(
    mgkit.snps.mapper.map_taxon_id_to_rank,
    taxonomy=tx,
    rank='genus'
)

filters = mgkit.snps.filter.get_default_filters(tx)

df = mgkit.snps.funcs.combine_sample_snps(
    a,
    min_num,
    filters,
    taxon_func=taxon_func,
    gene_func=None,
    index_type=None
)

dft = df.reorder_levels(['taxon', 'gene']).sortlevel('taxon')
```

```
INFO:mgkit.snps.funcs:Analysing SNP from sample t1_b3
2016-01-26 12:19:07,967 -    INFO - mgkit.snps.funcs->combine_sample_snps: Analysing SNP from sample t1_b3
INFO:mgkit.snps.funcs:Analysing SNP from sample t1_b2
2016-01-26 12:19:08,939 -    INFO - mgkit.snps.funcs->combine_sample_snps: Analysing SNP from sample t1_b2
INFO:mgkit.snps.funcs:Analysing SNP from sample t1_b1
2016-01-26 12:19:09,964 -    INFO - mgkit.snps.funcs->combine_sample_snps: Analysing SNP from sample t1_b1
INFO:mgkit.snps.funcs:Analysing SNP from sample t1_b7
2016-01-26 12:19:11,132 -    INFO - mgkit.snps.funcs->combine_sample_snps: Analysing SNP from sample t1_b7
INFO:mgkit.snps.funcs:Analysing SNP from sample t1_b6
2016-01-26 12:19:12,002 -    INFO - mgkit.snps.funcs->combine_sample_snps: Analysing SNP from sample t1_b6
INFO:mgkit.snps.funcs:Analysing SNP from sample t1_b5
2016-01-26 12:19:12,856 -    INFO - mgkit.snps.funcs->combine_sample_snps: Analysing SNP from sample t1_b5
INFO:mgkit.snps.funcs:Analysing SNP from sample t1_b4
2016-01-26 12:19:13,933 -    INFO - mgkit.snps.funcs->combine_sample_snps: Analysing SNP from sample t1_b4
INFO:mgkit.snps.funcs:Analysing SNP from sample t4_b1
2016-01-26 12:19:15,117 -    INFO - mgkit.snps.funcs->combine_sample_snps: Analysing SNP from sample t4_b1
INFO:mgkit.snps.funcs:Analysing SNP from sample t4_b2
2016-01-26 12:19:16,317 -    INFO - mgkit.snps.funcs->combine_sample_snps: Analysing SNP from sample t4_b2
INFO:mgkit.snps.funcs:Analysing SNP from sample t4_b3
2016-01-26 12:19:17,153 -    INFO - mgkit.snps.funcs->combine_sample_snps: Analysing SNP from sample t4_b3
INFO:mgkit.snps.funcs:Analysing SNP from sample t4_b4
2016-01-26 12:19:17,964 -    INFO - mgkit.snps.funcs->combine_sample_snps: Analysing SNP from sample t4_b4
INFO:mgkit.snps.funcs:Analysing SNP from sample t4_b5
2016-01-26 12:19:18,619 -    INFO - mgkit.snps.funcs->combine_sample_snps: Analysing SNP from sample t4_b5
INFO:mgkit.snps.funcs:Analysing SNP from sample t4_b6
2016-01-26 12:19:19,418 -    INFO - mgkit.snps.funcs->combine_sample_snps: Analysing SNP from sample t4_b6
INFO:mgkit.snps.funcs:Analysing SNP from sample t4_b7
2016-01-26 12:19:20,433 -    INFO - mgkit.snps.funcs->combine_sample_snps: Analysing SNP from sample t4_b7
```

## Define Constants¶

In [6]:

```
# Taxon ID for the most abundant genera
prev_id = 838
clos_id = 1485
meth_id = 2172

# Colours
clos_col = '#E41A1C'
prev_col = '#377EB8'
meth_col = '#4DAF4A'
comm_col = '#984EA3'
taxa_colors = {
    clos_id: clos_col,
    prev_id: prev_col,
    meth_id: meth_col
}
```

In [7]:

```
# Sets for each genus
clos_genes = dft.loc[clos_id].index
prev_genes = dft.loc[prev_id].index
meth_genes = dft.loc[meth_id].index
# Common genes
cp_genes = clos_genes & prev_genes
cm_genes = clos_genes & meth_genes
pm_genes = prev_genes & meth_genes
cpm_genes = clos_genes & prev_genes & meth_genes

common_genes = cp_genes | cm_genes | pm_genes | cpm_genes

# Found only in one genus
clos_only = clos_genes.difference(common_genes)
prev_only = prev_genes.difference(common_genes)
meth_only = meth_genes.difference(common_genes)
```

## Figure - Venn and Pie Charts¶

In [84]:

```
#figure - venn diagram (3-way), pie charts
fig = plt.figure(figsize=(13, 8), dpi=300)

gs_p = GridSpec(4, 2, width_ratios=[3, 1], height_ratios=[1, 1, 1, 0.25], hspace=0.25, wspace=.5)

# Genes in common between two taxa
data_test = (
    (cp_genes, clos_id, prev_id),
    (cm_genes, clos_id, meth_id),
    (pm_genes, prev_id, meth_id)
)

for ss, (genes, taxon_id1, taxon_id2) in zip(range(3), data_test):
    tmp_pval = {}
    # For each genes test if the distribution between the two taxa
    # is significantly different
    for gene in genes:
        tx1_vals = df.loc[gene].loc[taxon_id1].dropna()
        tx2_vals = df.loc[gene].loc[taxon_id2].dropna()
        pvalue = ranksums(tx1_vals, tx2_vals)[1] # Keep only the pvalue
        tmp_pval[gene] = pvalue
    # Corrects the pvalues with BH
    corr = r_func.correct_pvalues(pandas.Series(tmp_pval), method='BH')

    # Counts for the genes that are significantly different
    # how many have an higher pN/pS in one or the other
    tx1_genes = 0
    tx2_genes = 0
    # adjusted P<0.1
    for gene, pvalue in corr[corr < 0.1].iteritems():
        tx1_mean = df.loc[gene].loc[taxon_id1].mean()
        tx2_mean = df.loc[gene].loc[taxon_id2].mean()
        taxon_high = max((taxon_id1, tx1_mean), (taxon_id2, tx2_mean), key=lambda x: x[1])[0]
        if taxon_id1 == taxon_high:
            tx1_genes += 1
        else:
            tx2_genes += 1

    # Makes a pie chart for the test
    ax = fig.add_subplot(gs_p[ss, 1])
    # makes the taxa with the highest number a bit separate
    if tx1_genes > tx2_genes:
        explode = (0.2, 0.0, 0)
    elif tx2_genes > tx1_genes:
        explode = (0.0, 0.2, 0)
    else:
        explode = (0.0, 0.0, 0)
    # Makes the pie chart
    patches, texts = ax.pie(
        # numbers
        (tx1_genes, tx2_genes, len(corr[corr >= 0.1])),
        # factors for separation of the slice from the centre
        explode=explode,
        # Colours
        colors=(
            taxa_colors[taxon_id1],
            taxa_colors[taxon_id2],
            comm_col
        ),
        # Labels
        labels=(
#             "{} ({})".format(
#                 tx[taxon_id1].s_name.capitalize(),
#                 tx1_genes
#             ),
#             "{} ({})".format(
#                 tx[taxon_id2].s_name.capitalize(),
#                 tx2_genes
#             ),
#             'Non significant ({})'.format(len(corr[corr >= 0.1]))
            tx[taxon_id1].s_name.capitalize(),
            tx[taxon_id2].s_name.capitalize(),
            "Non significant"
        ),
        # To not use a shadow
        shadow=False,
        labeldistance=1.2,
        startangle=120
    )
    for text in texts[:-1]:
        text.set_style('italic')
    for text in texts:
        text.set_fontsize(14)
    # this makes the axes have the same proportions
    # can be also set add_subplot -> aspect='equal'
    ax.axis('equal')
    # Title of the chart
#     ax.set_title(
#         "Genes shared between\n{} and {}".format(
#             tx[taxon_id1].s_name.capitalize(),
#             tx[taxon_id2].s_name.capitalize()
#         ),
#         loc='center',
#         fontsize=16
#     )

# ax.text(1, 1, 'B', fontsize=32, ha='right')

vax = fig.add_subplot(gs_p[0:-1, 0])

# Makes the 3-way Venn diagram
venn_dg = venn3(
    # Gives the sets (they are pandas Indexes, convert)
    [set(clos_genes), set(prev_genes), set(meth_genes)],
    # labels
    set_labels=(
        tx[clos_id].s_name.capitalize(),
        tx[prev_id].s_name.capitalize(),
        tx[meth_id].s_name.capitalize()
    ),
    # Colors for each taxon
    set_colors=(clos_col, prev_col, meth_col),
    # Sets axis to span one column, 3 rows
    ax=vax,
)

# vax.text(vax.get_xlim()[0] + .5, vax.get_ylim()[1] * .5, 'A', fontsize=32, transform=vax.transAxes)

for text in venn_dg.set_labels:
    text.set_fontstyle('italic')
    text.set_fontsize(22)

for text in venn_dg.subset_labels:
    text.set_fontsize(18)

tax = fig.add_subplot(gs_p[3, 0])
tax.set_axis_off()
tax.text(.5, .5, 'A', fontsize=32, ha='center', va='center')

tax = fig.add_subplot(gs_p[3, 1])
tax.set_axis_off()
tax.text(.5, .5, 'B', fontsize=32, ha='center', va='center')
    
# Change layout
# fig.tight_layout(h_pad=2, rect=(0, 0, 0.9, 1))
fig.savefig('venn3-pie-min3.pdf', bbox_inches='tight')
```
