# Supplementary material for: Divergent functional isoforms drive niche specialisation for nutrient acquisition and use in rumen microbiome
Source: ISME J. 2017 Jan 13;11(4):932–44. doi: 10.1038/ismej.2016.172 (PMC5364355; doi:10.1038/ismej.2016.172)
Supplement: Supplementary File 5 [file ismej2016172x14.html]

EC stats-Final


# Enzyme Mappings¶

## Imports and Data Load¶

In [1]:

```
from __future__ import division
# Standard library imports
import textwrap
import cPickle as pickle

# Other libraries imports
import seaborn as sns
import matplotlib.pyplot as plt
import pandas as pd
import scipy.stats
import itertools
import pyperclip

# MGKit imports
import mgkit
import mgkit.kegg
import mgkit.plots.boxplot
from mgkit.mappings import enzyme
import mgkit.utils.r_func
from mgkit.snps.funcs import order_ratios
from mgkit.plots import boxplot_dataframe, get_single_figure, boxplot_dataframe_multindex, get_grid_figure
from mgkit.snps.conv_func import get_rank_dataframe, get_full_dataframe, get_gene_taxon_dataframe, get_gene_map_dataframe
from mgkit.utils.dictionary import apply_func_to_values
```

```
/Users/frubino/Dev/mgkit/dev-env/lib/python2.7/site-packages/matplotlib/__init__.py:872: UserWarning: axes.color_cycle is deprecated and replaced with axes.prop_cycle; please use the latter.
  warnings.warn(self.msg_depr % (key, alt_key))
```

In [2]:

```
# Configure logging
mgkit.logger.config_log()
```

In [3]:

```
#Load necessary data
snp_data = pickle.load(open('new_rfi_set.pickle', 'rb')) # SNPs data
taxonomy = mgkit.taxon.UniprotTaxonomy('data/taxonomy_full.pickle') # Taxononmy
```

```
INFO:mgkit.taxon:Loading taxonomy from file data/taxonomy_full.pickle
2016-01-26 00:02:30,028 -    INFO - mgkit.taxon->load_data: Loading taxonomy from file data/taxonomy_full.pickle
```

## Download KO to EC Mapping¶

The gene data in this study used Kegg Orthologs (KO) identifiers as *gene\_id* in the GFF and SNPs data. To avoid downloading mapping for all genes, because only a subset will be used, we can first build the dataframe and keep the list of KO identifiers.

In [4]:

```
df = get_full_dataframe(snp_data, taxonomy, index_type=None)
genes = set(df.index.get_level_values('gene'))
```

```
INFO:mgkit.snps.funcs:Analysing SNP from sample t1_b3
2016-01-26 00:02:38,998 -    INFO - mgkit.snps.funcs->combine_sample_snps: Analysing SNP from sample t1_b3
INFO:mgkit.snps.funcs:Analysing SNP from sample t1_b2
2016-01-26 00:02:40,063 -    INFO - mgkit.snps.funcs->combine_sample_snps: Analysing SNP from sample t1_b2
INFO:mgkit.snps.funcs:Analysing SNP from sample t1_b1
2016-01-26 00:02:41,287 -    INFO - mgkit.snps.funcs->combine_sample_snps: Analysing SNP from sample t1_b1
INFO:mgkit.snps.funcs:Analysing SNP from sample t1_b7
2016-01-26 00:02:42,885 -    INFO - mgkit.snps.funcs->combine_sample_snps: Analysing SNP from sample t1_b7
INFO:mgkit.snps.funcs:Analysing SNP from sample t1_b6
2016-01-26 00:02:44,024 -    INFO - mgkit.snps.funcs->combine_sample_snps: Analysing SNP from sample t1_b6
INFO:mgkit.snps.funcs:Analysing SNP from sample t1_b5
2016-01-26 00:02:45,289 -    INFO - mgkit.snps.funcs->combine_sample_snps: Analysing SNP from sample t1_b5
INFO:mgkit.snps.funcs:Analysing SNP from sample t1_b4
2016-01-26 00:02:46,640 -    INFO - mgkit.snps.funcs->combine_sample_snps: Analysing SNP from sample t1_b4
INFO:mgkit.snps.funcs:Analysing SNP from sample t4_b1
2016-01-26 00:02:48,022 -    INFO - mgkit.snps.funcs->combine_sample_snps: Analysing SNP from sample t4_b1
INFO:mgkit.snps.funcs:Analysing SNP from sample t4_b2
2016-01-26 00:02:49,335 -    INFO - mgkit.snps.funcs->combine_sample_snps: Analysing SNP from sample t4_b2
INFO:mgkit.snps.funcs:Analysing SNP from sample t4_b3
2016-01-26 00:02:50,394 -    INFO - mgkit.snps.funcs->combine_sample_snps: Analysing SNP from sample t4_b3
INFO:mgkit.snps.funcs:Analysing SNP from sample t4_b4
2016-01-26 00:02:51,378 -    INFO - mgkit.snps.funcs->combine_sample_snps: Analysing SNP from sample t4_b4
INFO:mgkit.snps.funcs:Analysing SNP from sample t4_b5
2016-01-26 00:02:52,190 -    INFO - mgkit.snps.funcs->combine_sample_snps: Analysing SNP from sample t4_b5
INFO:mgkit.snps.funcs:Analysing SNP from sample t4_b6
2016-01-26 00:02:53,118 -    INFO - mgkit.snps.funcs->combine_sample_snps: Analysing SNP from sample t4_b6
INFO:mgkit.snps.funcs:Analysing SNP from sample t4_b7
2016-01-26 00:02:54,351 -    INFO - mgkit.snps.funcs->combine_sample_snps: Analysing SNP from sample t4_b7
```

In [8]:

```
# MGKit as a class that can be used to access the Kegg REST interface
# The return value of the link_ids method is a dictionary KOs -> [EC_a, EC_b, .. EC_n]
# as each KO can map to multiple ECs
try:
    # saved ones for cache and make sure they are the same
    eclinks = pickle.load(open('eclinks.pickle', 'r')) 
except IOError:
    kc = mgkit.kegg.KeggClientRest()  # Init class
    eclinks = kc.link_ids('ec', list(genes)) # Request mappings from KOs to ECs
    pickle.dump(eclinks, open('eclinks.pickle', 'w'))
```

## Create DataFrames at Different EC Levels¶

In [9]:

```
# The mapped data must be transformed to a different specificity level
# The function used applies a simple transformation
eclinks1 = apply_func_to_values(eclinks, lambda x: x.split('.', 1)[0])
eclinks2 = apply_func_to_values(eclinks, lambda x: x.rsplit('.', 2)[0])
eclinks3 = apply_func_to_values(eclinks, lambda x: x.rsplit('.', 1)[0])
```

In [10]:

```
# This can be used to make dataframes at different level of specificity
# and the dataframe will show the diversity of each ECs among all organisms
# ecdf1 = get_gene_map_dataframe(snp_data, taxonomy, eclinks1)
ecdf2 = get_gene_map_dataframe(snp_data, taxonomy, eclinks2)
# ecdf2 = get_gene_map_dataframe(snp_data, taxonomy, eclinks3)
```

```
INFO:mgkit.snps.funcs:Analysing SNP from sample t1_b3
2016-01-26 00:06:24,823 -    INFO - mgkit.snps.funcs->combine_sample_snps: Analysing SNP from sample t1_b3
INFO:mgkit.snps.funcs:Analysing SNP from sample t1_b2
2016-01-26 00:06:25,227 -    INFO - mgkit.snps.funcs->combine_sample_snps: Analysing SNP from sample t1_b2
INFO:mgkit.snps.funcs:Analysing SNP from sample t1_b1
2016-01-26 00:06:25,616 -    INFO - mgkit.snps.funcs->combine_sample_snps: Analysing SNP from sample t1_b1
INFO:mgkit.snps.funcs:Analysing SNP from sample t1_b7
2016-01-26 00:06:25,984 -    INFO - mgkit.snps.funcs->combine_sample_snps: Analysing SNP from sample t1_b7
INFO:mgkit.snps.funcs:Analysing SNP from sample t1_b6
2016-01-26 00:06:26,355 -    INFO - mgkit.snps.funcs->combine_sample_snps: Analysing SNP from sample t1_b6
INFO:mgkit.snps.funcs:Analysing SNP from sample t1_b5
2016-01-26 00:06:26,648 -    INFO - mgkit.snps.funcs->combine_sample_snps: Analysing SNP from sample t1_b5
INFO:mgkit.snps.funcs:Analysing SNP from sample t1_b4
2016-01-26 00:06:27,109 -    INFO - mgkit.snps.funcs->combine_sample_snps: Analysing SNP from sample t1_b4
INFO:mgkit.snps.funcs:Analysing SNP from sample t4_b1
2016-01-26 00:06:27,604 -    INFO - mgkit.snps.funcs->combine_sample_snps: Analysing SNP from sample t4_b1
INFO:mgkit.snps.funcs:Analysing SNP from sample t4_b2
2016-01-26 00:06:28,004 -    INFO - mgkit.snps.funcs->combine_sample_snps: Analysing SNP from sample t4_b2
INFO:mgkit.snps.funcs:Analysing SNP from sample t4_b3
2016-01-26 00:06:28,306 -    INFO - mgkit.snps.funcs->combine_sample_snps: Analysing SNP from sample t4_b3
INFO:mgkit.snps.funcs:Analysing SNP from sample t4_b4
2016-01-26 00:06:28,592 -    INFO - mgkit.snps.funcs->combine_sample_snps: Analysing SNP from sample t4_b4
INFO:mgkit.snps.funcs:Analysing SNP from sample t4_b5
2016-01-26 00:06:28,820 -    INFO - mgkit.snps.funcs->combine_sample_snps: Analysing SNP from sample t4_b5
INFO:mgkit.snps.funcs:Analysing SNP from sample t4_b6
2016-01-26 00:06:29,098 -    INFO - mgkit.snps.funcs->combine_sample_snps: Analysing SNP from sample t4_b6
INFO:mgkit.snps.funcs:Analysing SNP from sample t4_b7
2016-01-26 00:06:29,447 -    INFO - mgkit.snps.funcs->combine_sample_snps: Analysing SNP from sample t4_b7
```

In [11]:

```
# a boxplot to show the diversty of ECs

sns.set_style('whitegrid')

fig, ax = mgkit.plots.get_single_figure(figsize=(40, 10), dpi=300)
_ = mgkit.plots.boxplot.boxplot_dataframe(
    ecdf2, 
    ecdf2.median(axis=1).sort(ascending=True, 
    inplace=False).index, 
    ax
)
ax.set_ylim(top=6)
```

```
/Users/frubino/Dev/mgkit/dev-env/lib/python2.7/site-packages/ipykernel/__main__.py:9: FutureWarning: sort is deprecated, use sort_values(inplace=True) for for INPLACE sorting
```

Out[11]:

```
(0.0, 6)
```

## Test Clostridium and Prevotella at EC Level 2¶

In [12]:

```
# Stores the taxon_id of Prevotella and Clostridium
clos_id = taxonomy.find_by_name('clostridium')[0]
prev_id = taxonomy.find_by_name('prevotella')[0]
```

```
DEBUG:mgkit.taxon:Generate name map
2016-01-26 00:06:46,809 -   DEBUG - mgkit.taxon->gen_name_map: Generate name map
```

In [13]:

```
# Make a dataframe with the a MultiIndex, where the key is (EC, taxon_id)
# To be stricter, we use only use genes in a minimum of seven samples
ecdfg = get_gene_taxon_dataframe(
    snp_data,  # SNPs data from snp_parser
    taxonomy,  # taxonomy
    eclinks2,  # the dictionary of KO->Mapping
    min_num=7  # minimum number of sample
)
```

```
INFO:mgkit.snps.funcs:Analysing SNP from sample t1_b3
2016-01-26 00:06:52,120 -    INFO - mgkit.snps.funcs->combine_sample_snps: Analysing SNP from sample t1_b3
INFO:mgkit.snps.funcs:Analysing SNP from sample t1_b2
2016-01-26 00:06:52,640 -    INFO - mgkit.snps.funcs->combine_sample_snps: Analysing SNP from sample t1_b2
INFO:mgkit.snps.funcs:Analysing SNP from sample t1_b1
2016-01-26 00:06:53,213 -    INFO - mgkit.snps.funcs->combine_sample_snps: Analysing SNP from sample t1_b1
INFO:mgkit.snps.funcs:Analysing SNP from sample t1_b7
2016-01-26 00:06:53,808 -    INFO - mgkit.snps.funcs->combine_sample_snps: Analysing SNP from sample t1_b7
INFO:mgkit.snps.funcs:Analysing SNP from sample t1_b6
2016-01-26 00:06:54,268 -    INFO - mgkit.snps.funcs->combine_sample_snps: Analysing SNP from sample t1_b6
INFO:mgkit.snps.funcs:Analysing SNP from sample t1_b5
2016-01-26 00:06:54,729 -    INFO - mgkit.snps.funcs->combine_sample_snps: Analysing SNP from sample t1_b5
INFO:mgkit.snps.funcs:Analysing SNP from sample t1_b4
2016-01-26 00:06:55,362 -    INFO - mgkit.snps.funcs->combine_sample_snps: Analysing SNP from sample t1_b4
INFO:mgkit.snps.funcs:Analysing SNP from sample t4_b1
2016-01-26 00:06:56,030 -    INFO - mgkit.snps.funcs->combine_sample_snps: Analysing SNP from sample t4_b1
INFO:mgkit.snps.funcs:Analysing SNP from sample t4_b2
2016-01-26 00:06:56,615 -    INFO - mgkit.snps.funcs->combine_sample_snps: Analysing SNP from sample t4_b2
INFO:mgkit.snps.funcs:Analysing SNP from sample t4_b3
2016-01-26 00:06:57,075 -    INFO - mgkit.snps.funcs->combine_sample_snps: Analysing SNP from sample t4_b3
INFO:mgkit.snps.funcs:Analysing SNP from sample t4_b4
2016-01-26 00:06:57,506 -    INFO - mgkit.snps.funcs->combine_sample_snps: Analysing SNP from sample t4_b4
INFO:mgkit.snps.funcs:Analysing SNP from sample t4_b5
2016-01-26 00:06:57,874 -    INFO - mgkit.snps.funcs->combine_sample_snps: Analysing SNP from sample t4_b5
INFO:mgkit.snps.funcs:Analysing SNP from sample t4_b6
2016-01-26 00:06:58,284 -    INFO - mgkit.snps.funcs->combine_sample_snps: Analysing SNP from sample t4_b6
INFO:mgkit.snps.funcs:Analysing SNP from sample t4_b7
2016-01-26 00:06:58,820 -    INFO - mgkit.snps.funcs->combine_sample_snps: Analysing SNP from sample t4_b7
```

In [14]:

```
# The index is in the form (EC, taxon_id). To access the data using taxon_id as key
# the index must be reordered and the level sorted
ecdfg = ecdfg.reorder_levels(['taxon', 'gene']).sortlevel('taxon')
```

In [15]:

```
# the file "enzclass.txt" can be download from expasy and contains
# description for each enzyme class, the function parses it into a
# dictionary
enzclass_file = 'enzclass.txt'
labels = enzyme.parse_expasy_file(enzclass_file)
# We can also update the content with descriptions from Kegg
labels.update(mgkit.kegg.KeggClientRest().get_names('ec'))
```

In [16]:

```
# Tests all genes in common between Prevotella and Clostridium
# using the ranksums test implementation in SciPy
pvalues = {}
for ec_id in ecdfg.loc[clos_id].index & ecdfg.loc[prev_id].index:
    clos_vals = ecdfg.loc[clos_id].loc[ec_id]
    prev_vals = ecdfg.loc[prev_id].loc[ec_id]
    pvalues[ec_id] = scipy.stats.ranksums(clos_vals, prev_vals)[1] # Only the pvalue is kept

pvalues = pd.Series(pvalues)  # create a pandas Series

# This function used R to correct the pvalues. The function requires a pandas Series
corr = mgkit.utils.r_func.correct_pvalues(pvalues, method='BH')  # Use BH for correction
```

In [17]:

```
# Quick check
len(pvalues), len(pvalues[pvalues < 0.05]), len(corr[corr < 0.05])
```

Out[17]:

```
(34, 22, 21)
```

In [18]:

```
# Create a new dataframe with only the genes that were significantly
# different at a adjusted P<0.05
common_genes_sign = ecdfg.select(
    lambda x: (x[0] in [clos_id, prev_id]) and (x[1] in corr[corr < 0.05].index)
).reorder_levels(['gene', 'taxon'])
# Reorder the index to use the the gene_id as first key
common_genes_sign.sortlevel('gene', inplace=True)
```

In [19]:

```
# Determines which significant gene has a higher mean pN/pS in Prevotella
# or Clostridium
genes_higher = common_genes_sign.mean(axis=1).groupby(
    level='gene').aggregate(lambda x: 'P' if x[0] > x[1] else 'C'
)

clos_higher = common_genes_sign.loc[list(genes_higher[genes_higher == 'C'].index)]
prev_higher = common_genes_sign.loc[list(genes_higher[genes_higher == 'P'].index)]
```

### Table¶

In [20]:

```
# Makes the table for publication
table_ec = ecdfg.loc[[clos_id, prev_id]].swaplevel(0, 1).sortlevel(0).loc[list(corr[corr < 0.05].index)]
table_ec = pd.DataFrame({
    ('Number of Samples', ''): table_ec.count(axis=1),
    ('pN/pS', 'Mean'): table_ec.mean(axis=1),
    ('pN/pS', 'Std'): table_ec.std(axis=1),
    ('pN/pS', 'Min'): table_ec.min(axis=1),
    ('pN/pS', 'Max'): table_ec.max(axis=1),
}).rename(
    index=lambda x: '[EC ' + x + '.-] ' + labels[x] if isinstance(x, str) else taxonomy[x].s_name.capitalize()
)
table_ec.index.names = ['Enzyme Class', 'Genus']
```

In [21]:

```
#CSV
table_ec[
    [('Number of Samples', ''), ('pN/pS', 'Mean'), ('pN/pS', 'Std'), ('pN/pS', 'Min'), ('pN/pS', 'Max')]
].to_csv('ec_genus.csv', float_format="%.2f")
```

### Figures¶

In [64]:

```
# Creates a 3x2 grid figure for EC that are significantly different and
# have higher mean pN/pS in Prevotella
fig, gs = get_grid_figure(2, 3, figsize=(15, 15), dpi=300, wspace=0.25, hspace=0.25)

# The order is chosen to better group the data
for gsid, ec_id in enumerate(['1.1', '3.4', '1.2', '4.99', '1.4', '5.3']):
    ax = plt.subplot(gs[gsid])  # Init the axis
    boxplot_dataframe(
        prev_higher.loc[ec_id],  # returns the EC class with the values for Prevotella/Clostridium
        [prev_id, clos_id],  # the order (left from right) in which the boxplot are to be drawn
        ax,  # Axes instance
        label_map={clos_id: 'Clostridium', prev_id: 'Prevotella'},  # Dictionary for the labels on the X axis
        data_colours={clos_id: '#E41A1C', prev_id: '#377EB8'},  # Colours to be used
        fonts={'rotation': 0, 'fontsize': 22},  # Font options
        widths=0.3  # boxplot width
    )
    # Set the boxplot for the axes.
    ax.set_title(
                "[EC {}.-]\n({})\n{}".format(
                    ec_id, 
                    labels[ec_id.split('.')[0]], 
                    '\n'.join(textwrap.wrap(labels[ec_id], 30)))
                ,
        fontsize=18
    )
    # Set the grid only on the pN/pS axis
    ax.grid(axis='x')
#     for text in ax.get_xticklabels():
#         text.set_style('italic')
        #text.set_weight('bold')
    ax.set_xticklabels([])
# Save figure
fig.savefig('ec-plot-sign-prev-l2.pdf', bbox_inches='tight')
```

In [76]:

```
# Creates a 3x5 grid figure for EC that are significantly different and
# have higher mean pN/pS in Prevotella
NCOL = 5
NROW = 3
fig, gs = get_grid_figure(NROW, NCOL, figsize=(NCOL * 5, NROW * 5 - 1), wspace=0.25, hspace=0.40)

for gsid, ec_id in enumerate(['1.3', '1.6', '3.2', '3.5', '3.7', '2.2', '2.3', '2.4', '2.7', '4.1', '5.1', '5.2', '6.1', '6.2', '6.6']):
    ax = plt.subplot(gs[gsid])
    boxplot_dataframe(
        clos_higher.loc[ec_id], # returns the EC class with the values for Prevotella/Clostridium
        [prev_id, clos_id],  # the order (left from right) in which the boxplot are to be drawn
        ax,  # Axes instance
        label_map={clos_id: 'Clostridium', prev_id: 'Prevotella'},  # Dictionary for the labels on the X axis
        data_colours={clos_id: '#E41A1C', prev_id: '#377EB8'},  # Colours to be used
        fonts={'rotation': 0, 'fontsize': 21},  # Font options
        widths=0.3  # boxplot width
    )
    # Set the boxplot for the axes.
    ax.set_title(
#         "[EC {}.-]\n({})\n{}".format(
        "[EC {}.-]\n{}".format(
            ec_id, 
#             labels[ec_id.split('.')[0]], 
            '\n'.join(textwrap.wrap(labels[ec_id], 20 if ec_id in ['1.6', '5.1'] else 25))
        ),
        fontsize=22
    )
    # Set the grid only on the pN/pS axis
    ax.grid(axis='x')
#     for text in ax.get_xticklabels():
#         text.set_style('italic')
        #text.set_weight('bold')
    ax.set_xticklabels([])
# Save figure
fig.savefig('ec-plot-sign-clos-l2.pdf', bbox_inches='tight')
```

## Overrepresentation Analysis¶

In [ ]:

```
# Creates the background for analysis from all genes
# that are in Prevotella and Clostridium and put
# them in two sets
clos_genes = set()
prev_genes = set()
for genesnp in snp_data['t1_b1'].itervalues():
    if taxonomy.is_ancestor(genesnp.taxon_id, clos_id):
        clos_genes.add(genesnp.gene_id.split('.')[0])
    elif taxonomy.is_ancestor(genesnp.taxon_id, prev_id):
        prev_genes.add(genesnp.gene_id.split('.')[0])
```

In [ ]:

```
# Gets the mapping for these genes
ko2ec = kc.link_ids('ec', list(clos_genes | prev_genes))
```

In [ ]:

```
# Reverses the mapping, making a dictionary EC -> KO
ec2ko = mgkit.utils.dictionary.reverse_mapping(ko2ec)
```

In [ ]:

```
# Transforms the keys to EC of level 2 specificity
ec2ko2 = {}
for ec_id, gene_ids in ec2ko.iteritems():
    ec_id = enzyme.get_enzyme_level(ec_id, 2)
    try:
        ec2ko2[ec_id].update(gene_ids)
    except KeyError:
        ec2ko2[ec_id] = set(gene_ids)
```

In [ ]:

```
# Makes a dataframe with boolean values for all significantly
# different genes. if the value is True, that gene has a higher
# pN/pS in Clostridium
ecsign = ecdfg.loc[clos_id].loc[corr[corr < 0.05].index].mean(axis=1) > ecdfg.loc[prev_id].loc[corr[corr < 0.05].index].mean(axis=1)
```

In [ ]:

```
# Saves files for analysis with GOSeq
# Foreground for Clostridium
f = open('clos-sign.txt', 'w')
# Foreground for Prevotella
g = open('prev-sign.txt', 'w')
for ec_id, sign in ecsign.iterkv():
    gene_ids = ec2ko2[ec_id]
    if sign:
        f.write('\n'.join(clos_genes & gene_ids))        
        f.write('n')
    else:
        g.write('\n'.join(prev_genes & gene_ids))
        g.write('n')
f.close()
g.close()
```

In [ ]:

```
# Mapping KO to EC
with open('ko-ec-map.txt', 'w') as f:
    f.write('ID\tPathway\n')
    for ec_id, gene_ids in ec2ko2.iteritems():
        for gene_id in gene_ids:
            f.write("{}\t{}\n".format(gene_id, ec_id))
```

In [ ]:

```
# Background
with open('ec-background.txt', 'w') as f:
    for gene_id in clos_genes | prev_genes:
        f.write(gene_id + '\n')
```

In [ ]:

```
# After running GOSeq and the file saved to disk, it can be opened
# into a spreadsheet software. After copying the pvalue column to
# the clipboard, the pvalues can beadjusted with this line (they
# are copied back adjusted to the clipboard)
mgkit.utils.r_func.correct_pvalues(pd.Series(float(x) for x in pyperclip.paste().split())).to_clipboard()
```
