# Supplementary material for: Divergent functional isoforms drive niche specialisation for nutrient acquisition and use in rumen microbiome
Source: ISME J. 2017 Jan 13;11(4):932–44. doi: 10.1038/ismej.2016.172 (PMC5364355; doi:10.1038/ismej.2016.172)
Supplement: Supplementary File 6 [file ismej2016172x15.html]

Pathway modules analysis-Paper


In [1]:

```
import itertools
import cPickle as pickle

import numpy
import pandas
import matplotlib.patches as mpatches
import seaborn as sns
import scipy.stats

import mgkit.utils.r_func
import mgkit.plots
import mgkit.kegg
import mgkit.taxon
import mgkit.snps
from mgkit.plots import boxplot_dataframe, get_single_figure, boxplot_dataframe_multindex
```

```
/Users/frubino/Dev/mgkit/dev-env/lib/python2.7/site-packages/matplotlib/__init__.py:872: UserWarning: axes.color_cycle is deprecated and replaced with axes.prop_cycle; please use the latter.
  warnings.warn(self.msg_depr % (key, alt_key))
```

In [2]:

```
import mgkit; mgkit.logger.config_log()
```

In [4]:

```
try:
    modules, ko_links = pickle.load(open('module-links.pickle', 'r'))
except IOError:
    kc = mgkit.kegg.KeggClientRest()
    modules = itertools.chain(*kc.link_ids('module', 'map01200').values())
    modules = {module: mgkit.kegg.KeggModule(kc.get_entry(module)) for module in modules}
    ko_links = kc.link_ids('ko', modules.keys())
    
    pickle.dump((modules, ko_links), open('module-links.pickle', 'w'))
```

In [5]:

```
a = pickle.load(open('new_rfi_set.pickle', 'rb'))
tx = mgkit.taxon.UniprotTaxonomy('data/taxonomy_full.pickle')
df = mgkit.snps.conv_func.get_rank_dataframe(a, tx, rank='genus', min_num=3, index_type=None)
```

```
INFO:mgkit.taxon:Loading taxonomy from file data/taxonomy_full.pickle
2016-01-26 11:24:40,720 -    INFO - mgkit.taxon->load_data: Loading taxonomy from file data/taxonomy_full.pickle
INFO:mgkit.snps.funcs:Analysing SNP from sample t1_b3
2016-01-26 11:24:48,971 -    INFO - mgkit.snps.funcs->combine_sample_snps: Analysing SNP from sample t1_b3
INFO:mgkit.snps.funcs:Analysing SNP from sample t1_b2
2016-01-26 11:24:49,747 -    INFO - mgkit.snps.funcs->combine_sample_snps: Analysing SNP from sample t1_b2
INFO:mgkit.snps.funcs:Analysing SNP from sample t1_b1
2016-01-26 11:24:50,615 -    INFO - mgkit.snps.funcs->combine_sample_snps: Analysing SNP from sample t1_b1
INFO:mgkit.snps.funcs:Analysing SNP from sample t1_b7
2016-01-26 11:24:51,647 -    INFO - mgkit.snps.funcs->combine_sample_snps: Analysing SNP from sample t1_b7
INFO:mgkit.snps.funcs:Analysing SNP from sample t1_b6
2016-01-26 11:24:52,422 -    INFO - mgkit.snps.funcs->combine_sample_snps: Analysing SNP from sample t1_b6
INFO:mgkit.snps.funcs:Analysing SNP from sample t1_b5
2016-01-26 11:24:53,181 -    INFO - mgkit.snps.funcs->combine_sample_snps: Analysing SNP from sample t1_b5
INFO:mgkit.snps.funcs:Analysing SNP from sample t1_b4
2016-01-26 11:24:54,147 -    INFO - mgkit.snps.funcs->combine_sample_snps: Analysing SNP from sample t1_b4
INFO:mgkit.snps.funcs:Analysing SNP from sample t4_b1
2016-01-26 11:24:55,189 -    INFO - mgkit.snps.funcs->combine_sample_snps: Analysing SNP from sample t4_b1
INFO:mgkit.snps.funcs:Analysing SNP from sample t4_b2
2016-01-26 11:24:56,179 -    INFO - mgkit.snps.funcs->combine_sample_snps: Analysing SNP from sample t4_b2
INFO:mgkit.snps.funcs:Analysing SNP from sample t4_b3
2016-01-26 11:24:56,919 -    INFO - mgkit.snps.funcs->combine_sample_snps: Analysing SNP from sample t4_b3
INFO:mgkit.snps.funcs:Analysing SNP from sample t4_b4
2016-01-26 11:24:57,601 -    INFO - mgkit.snps.funcs->combine_sample_snps: Analysing SNP from sample t4_b4
INFO:mgkit.snps.funcs:Analysing SNP from sample t4_b5
2016-01-26 11:24:58,166 -    INFO - mgkit.snps.funcs->combine_sample_snps: Analysing SNP from sample t4_b5
INFO:mgkit.snps.funcs:Analysing SNP from sample t4_b6
2016-01-26 11:24:58,846 -    INFO - mgkit.snps.funcs->combine_sample_snps: Analysing SNP from sample t4_b6
INFO:mgkit.snps.funcs:Analysing SNP from sample t4_b7
2016-01-26 11:24:59,742 -    INFO - mgkit.snps.funcs->combine_sample_snps: Analysing SNP from sample t4_b7
```

In [6]:

```
mod_names = kc.get_ids_names('module')
```

In [7]:

```
prev_id = 838
clos_id = 1485
clos_genes = df.select(lambda x: x[1] == clos_id).index.get_level_values('gene')
prev_genes = df.select(lambda x: x[1] == prev_id).index.get_level_values('gene')
dfc = df.reorder_levels(['taxon', 'gene']).loc[clos_id].loc[clos_genes]
dfp = df.reorder_levels(['taxon', 'gene']).loc[prev_id].loc[prev_genes]
```

In [8]:

```
cdata = pandas.DataFrame.from_dict({
    module: dfc.loc[ko_links[module]].unstack().dropna()
    for module in modules
    if set(ko_links[module]) & set(dfc.index)
}, orient='index')
pdata = pandas.DataFrame.from_dict({
    module: dfp.loc[ko_links[module]].unstack().dropna()
    for module in modules
    if set(ko_links[module]) & set(dfp.index)
}, orient='index')
```

In [9]:

```
pvalues = pandas.Series({module: scipy.stats.ranksums(cdata.loc[module].dropna(), pdata.loc[module].dropna())[1] for module in cdata.index & pdata.index})
corr = mgkit.utils.r_func.correct_pvalues(pvalues)

for module in corr[corr < 0.1].index:
    print module, mod_names[module], cdata.loc[module].dropna().mean(), pdata.loc[module].dropna().mean(), corr[module] < 0.1, pvalues[module]
```

```
M00005 PRPP biosynthesis, ribose 5P => PRPP 2.75851122381 0.130986279497 True 0.000348575174213
M00020 Serine biosynthesis, glycerate-3P => serine 5.08962065478 0.192109293383 True 0.0407013545561
M00021 Cysteine biosynthesis, serine => cysteine 1.63501917788 0.13019407348 True 0.000175626939456
M00167 Reductive pentose phosphate cycle, glyceraldehyde-3P => ribulose-5P 1.10153057371 0.601456364149 True 0.0270268067959
M00307 Pyruvate oxidation, pyruvate => acetyl-CoA 0.164797073781 1.50258263263 True 0.00142314434658
M00345 Formaldehyde assimilation, ribulose monophosphate pathway 1.89018254742 0.610762345825 True 0.000531727528354
M00346 Formaldehyde assimilation, serine pathway 0.745982241258 0.18293957693 True 1.44029225145e-06
M00373 Ethylmalonyl pathway 0.0111337406653 0.0460259349134 True 0.0175636857511
M00375 Hydroxypropionate-hydroxybutylate cycle 0.0111337406653 0.743990596212 True 0.00347001734451
M00377 Reductive acetyl-CoA pathway (Wood-Ljungdahl pathway) 0.0 0.347004872477 True 0.0313886643773
M00567 Methanogenesis, CO2 => methane 0.0 0.55833623954 True 0.00158763960477
M00741 Propanoyl-CoA metabolism, propanoyl-CoA => succinyl-CoA 0.0111337406653 0.542747937855 True 0.00519373714548
```

In [10]:

```
import itertools
multidx = pandas.MultiIndex.from_tuples(
    list(
        itertools.product(
            set(corr[corr < 0.1].index) - set(['M00567', 'M00357']), 
            [clos_id, prev_id])
    ), 
    names=['module', 'taxon']
)
```

In [11]:

```
cdata = pandas.DataFrame.from_dict({
    module : df.reorder_levels(['taxon', 'gene']).loc[clos_id].loc[ko_links[module]].unstack().dropna()
    for module in corr[corr < 0.1].index if module not in ['M00567', 'M00357']
    if set(ko_links[module]) & set(dfc.index)
}, orient='index')
pdata = pandas.DataFrame.from_dict({
    module: df.reorder_levels(['taxon', 'gene']).loc[prev_id].loc[ko_links[module]].unstack().dropna()
    for module in corr[corr < 0.1].index if module not in ['M00567', 'M00357']
    if set(ko_links[module]) & set(dfp.index)
}, orient='index')

adata = pandas.concat([cdata, pdata], axis=0, keys=[clos_id, prev_id]).reorder_levels([1,0])
adata.sortlevel(0, inplace=True)
```

In [22]:

```
sns.set_style('whitegrid')
#from mgkit.plots.unused import map_taxon_to_colours, TAXON_COLOURS
fig, ax = get_single_figure(dpi=400, figsize=(15, 19))
data_colors = {clos_id: '#E41A1C', prev_id: '#377EB8'}
import textwrap
mod_wrapped = {mod_id: '\n'.join(textwrap.wrap(mod_name, 30)) for mod_id, mod_name in mod_names.iteritems()}
_ = boxplot_dataframe_multindex(
    adata.rename(index=mod_wrapped), ax, fonts={'fontsize': 26, 'rotation': 0}, data_colours=data_colors, box_vert=False
)
ax.grid(axis='y')

patches = [
    mpatches.Patch(color=data_colors[clos_id], label='Clostridium'),
    mpatches.Patch(color=data_colors[prev_id], label='Prevotella'),
]
_ = ax.legend(handles=patches, fontsize=26, markerscale=22)
for text in _.get_texts(): text.set_style('italic')
ax.set_xlim(right=6)
ax.set_xlabel('pN/pS', fontsize=24)
ax.set_ylabel('Kegg Module', fontsize=24)
for xpos in numpy.arange(2.5, ax.get_ylim()[1], 2):
    ax.axhline(xpos, linestyle=':', color='k')
for spos, spine in ax.spines.iteritems():
    if spos == 'right':
        continue
    spine.set_visible(False)
#ax.set_xscale('symlog')
fig.tight_layout()
fig.savefig('modules-sign.pdf', bbox_inches='tight')
```
