# Supplementary material for: Divergent functional isoforms drive niche specialisation for nutrient acquisition and use in rumen microbiome
Source: ISME J. 2017 Jan 13;11(4):932–44. doi: 10.1038/ismej.2016.172 (PMC5364355; doi:10.1038/ismej.2016.172)
Supplement: Supplementary File 7 [file ismej2016172x16.html]

Taxa Variation-Final


# Taxa Variation¶

## Import and Data Load¶

In [1]:

```
from __future__ import division

# Standard library
import cPickle as pickle

# External libraries imports
import seaborn as sns
import matplotlib.pyplot as plt
import pandas as pd
import numpy as np
from matplotlib import patches as mpatches

# MGKit imports
import mgkit
from mgkit.snps.funcs import order_ratios
from mgkit.plots import boxplot_dataframe, get_single_figure
from mgkit.snps.conv_func import get_rank_dataframe
```

```
/Users/frubino/Dev/mgkit/dev-env/lib/python2.7/site-packages/matplotlib/__init__.py:872: UserWarning: axes.color_cycle is deprecated and replaced with axes.prop_cycle; please use the latter.
  warnings.warn(self.msg_depr % (key, alt_key))
```

In [2]:

```
# Log config
mgkit.logger.config_log()
```

In [4]:

```
# Loads SNPs data
snp_data = pickle.load(open('new_rfi_set.pickle', 'rb'))
```

In [5]:

```
# Loads taxonomy
taxonomy = mgkit.taxon.UniprotTaxonomy('data/taxonomy_full.pickle')
```

```
INFO:mgkit.taxon:Loading taxonomy from file data/taxonomy_full.pickle
2016-01-26 11:46:45,362 -    INFO - mgkit.taxon->load_data: Loading taxonomy from file data/taxonomy_full.pickle
```

## Table (Genera)¶

In [6]:

```
# Builds a dataframe for taxa, at the genus level
df = get_rank_dataframe(snp_data, taxonomy, rank='genus', index_type='taxon')
```

```
INFO:mgkit.snps.funcs:Analysing SNP from sample t1_b3
2016-01-26 11:46:54,509 -    INFO - mgkit.snps.funcs->combine_sample_snps: Analysing SNP from sample t1_b3
INFO:mgkit.snps.funcs:Analysing SNP from sample t1_b2
2016-01-26 11:46:55,082 -    INFO - mgkit.snps.funcs->combine_sample_snps: Analysing SNP from sample t1_b2
INFO:mgkit.snps.funcs:Analysing SNP from sample t1_b1
2016-01-26 11:46:55,624 -    INFO - mgkit.snps.funcs->combine_sample_snps: Analysing SNP from sample t1_b1
INFO:mgkit.snps.funcs:Analysing SNP from sample t1_b7
2016-01-26 11:46:56,140 -    INFO - mgkit.snps.funcs->combine_sample_snps: Analysing SNP from sample t1_b7
INFO:mgkit.snps.funcs:Analysing SNP from sample t1_b6
2016-01-26 11:46:56,593 -    INFO - mgkit.snps.funcs->combine_sample_snps: Analysing SNP from sample t1_b6
INFO:mgkit.snps.funcs:Analysing SNP from sample t1_b5
2016-01-26 11:46:56,987 -    INFO - mgkit.snps.funcs->combine_sample_snps: Analysing SNP from sample t1_b5
INFO:mgkit.snps.funcs:Analysing SNP from sample t1_b4
2016-01-26 11:46:57,583 -    INFO - mgkit.snps.funcs->combine_sample_snps: Analysing SNP from sample t1_b4
INFO:mgkit.snps.funcs:Analysing SNP from sample t4_b1
2016-01-26 11:46:58,236 -    INFO - mgkit.snps.funcs->combine_sample_snps: Analysing SNP from sample t4_b1
INFO:mgkit.snps.funcs:Analysing SNP from sample t4_b2
2016-01-26 11:46:58,786 -    INFO - mgkit.snps.funcs->combine_sample_snps: Analysing SNP from sample t4_b2
INFO:mgkit.snps.funcs:Analysing SNP from sample t4_b3
2016-01-26 11:46:59,174 -    INFO - mgkit.snps.funcs->combine_sample_snps: Analysing SNP from sample t4_b3
INFO:mgkit.snps.funcs:Analysing SNP from sample t4_b4
2016-01-26 11:46:59,546 -    INFO - mgkit.snps.funcs->combine_sample_snps: Analysing SNP from sample t4_b4
INFO:mgkit.snps.funcs:Analysing SNP from sample t4_b5
2016-01-26 11:46:59,867 -    INFO - mgkit.snps.funcs->combine_sample_snps: Analysing SNP from sample t4_b5
INFO:mgkit.snps.funcs:Analysing SNP from sample t4_b6
2016-01-26 11:47:00,256 -    INFO - mgkit.snps.funcs->combine_sample_snps: Analysing SNP from sample t4_b6
INFO:mgkit.snps.funcs:Analysing SNP from sample t4_b7
2016-01-26 11:47:00,733 -    INFO - mgkit.snps.funcs->combine_sample_snps: Analysing SNP from sample t4_b7
```

In [7]:

```
taxa_variation = df.copy()
taxa_variation.index.name = 'taxon_id'
taxa_variation['taxon_name'] = pd.Series({x: taxonomy[x].s_name for x in taxa_variation.index})
taxa_variation['lineage'] = pd.Series({x: ':'.join(taxonomy[x].lineage) for x in taxa_variation.index})
taxa_variation[[-2,-1] + range(0, 14)].to_csv('taxa_variation.csv')
```

## Figures¶

In [8]:

```
# Builds dataframe at genus lavel including genes
dfgenes = get_rank_dataframe(snp_data, taxonomy, rank='genus', index_type=None)
gene_counts = dfgenes.mean(axis=1).count(level=1)
gene_counts = gene_counts[gene_counts > 0]
```

```
INFO:mgkit.snps.funcs:Analysing SNP from sample t1_b3
2016-01-26 11:47:14,628 -    INFO - mgkit.snps.funcs->combine_sample_snps: Analysing SNP from sample t1_b3
INFO:mgkit.snps.funcs:Analysing SNP from sample t1_b2
2016-01-26 11:47:15,557 -    INFO - mgkit.snps.funcs->combine_sample_snps: Analysing SNP from sample t1_b2
INFO:mgkit.snps.funcs:Analysing SNP from sample t1_b1
2016-01-26 11:47:16,537 -    INFO - mgkit.snps.funcs->combine_sample_snps: Analysing SNP from sample t1_b1
INFO:mgkit.snps.funcs:Analysing SNP from sample t1_b7
2016-01-26 11:47:17,712 -    INFO - mgkit.snps.funcs->combine_sample_snps: Analysing SNP from sample t1_b7
INFO:mgkit.snps.funcs:Analysing SNP from sample t1_b6
2016-01-26 11:47:18,596 -    INFO - mgkit.snps.funcs->combine_sample_snps: Analysing SNP from sample t1_b6
INFO:mgkit.snps.funcs:Analysing SNP from sample t1_b5
2016-01-26 11:47:19,398 -    INFO - mgkit.snps.funcs->combine_sample_snps: Analysing SNP from sample t1_b5
INFO:mgkit.snps.funcs:Analysing SNP from sample t1_b4
2016-01-26 11:47:20,460 -    INFO - mgkit.snps.funcs->combine_sample_snps: Analysing SNP from sample t1_b4
INFO:mgkit.snps.funcs:Analysing SNP from sample t4_b1
2016-01-26 11:47:21,611 -    INFO - mgkit.snps.funcs->combine_sample_snps: Analysing SNP from sample t4_b1
INFO:mgkit.snps.funcs:Analysing SNP from sample t4_b2
2016-01-26 11:47:22,746 -    INFO - mgkit.snps.funcs->combine_sample_snps: Analysing SNP from sample t4_b2
INFO:mgkit.snps.funcs:Analysing SNP from sample t4_b3
2016-01-26 11:47:23,607 -    INFO - mgkit.snps.funcs->combine_sample_snps: Analysing SNP from sample t4_b3
INFO:mgkit.snps.funcs:Analysing SNP from sample t4_b4
2016-01-26 11:47:24,396 -    INFO - mgkit.snps.funcs->combine_sample_snps: Analysing SNP from sample t4_b4
INFO:mgkit.snps.funcs:Analysing SNP from sample t4_b5
2016-01-26 11:47:25,028 -    INFO - mgkit.snps.funcs->combine_sample_snps: Analysing SNP from sample t4_b5
INFO:mgkit.snps.funcs:Analysing SNP from sample t4_b6
2016-01-26 11:47:25,781 -    INFO - mgkit.snps.funcs->combine_sample_snps: Analysing SNP from sample t4_b6
INFO:mgkit.snps.funcs:Analysing SNP from sample t4_b7
2016-01-26 11:47:26,766 -    INFO - mgkit.snps.funcs->combine_sample_snps: Analysing SNP from sample t4_b7
```

In [9]:

```
# Finds the number of genes per taxa that cover at least
# 55% of the total. This is done to create a smaller figure
min_genes = 0
for x in range(1, gene_counts.max())[::-1]:
    info = gene_counts[gene_counts >= x].sum() / gene_counts.sum()
    if info >= .55:
        min_genes = x
        print x, info
        break
```

```
93 0.550553045646
```

In [10]:

```
# Gets the color for each taxonomic group
def get_ancestor_color(taxonomy, plot_order):
    plot_colors = {}
    for taxon_id in plot_order:
        if taxonomy.is_ancestor(taxon_id, mgkit.taxon.ARCHAEA):
            plot_colors[taxon_id] = sns.color_palette('Set1', 4)[2]
        elif taxonomy.is_ancestor(taxon_id, mgkit.taxon.BACTERIA):
            plot_colors[taxon_id] = sns.color_palette('Set1', 4)[1]
        elif taxonomy.is_ancestor(taxon_id, mgkit.taxon.FUNGI):
            plot_colors[taxon_id] = sns.color_palette('Set1', 4)[0]
        elif taxonomy.is_ancestor(taxon_id, mgkit.taxon.PROTISTS.values()):
            plot_colors[taxon_id] = sns.color_palette('Set1', 4)[3]
    return plot_colors
```

In [11]:

```
# Select the taxa that the minimum required number of genes
taxon_ids = gene_counts[gene_counts >= min_genes].index

# Creates label map
label_map = {x: "{} ({})".format(taxonomy[x].s_name, gene_counts[x]) for x in taxon_ids}
# Assign colours
plot_colors = get_ancestor_color(taxonomy, taxon_ids)
```

In [12]:

```
# Makes distance matrix for taxa, based on their taxonomic distance
groups = mgkit.taxon.taxa_distance_matrix(taxonomy, taxon_ids)
```

In [26]:

```
sns.set_style('whitegrid')
# Figure grid 1x3
fig, gs = mgkit.plots.get_grid_figure(
    1, 
    3, 
    figsize=(20, 8), 
    width_ratios=[1, 2, 1],
    wspace=0.0, 
    hspace=0
)

# dendrogram on the left
ax = fig.add_subplot(gs[0, 0])

# plots the dendrogram
d_data = mgkit.plots.heatmap.dendrogram(groups, ax, orientation='right', method='complete', use_dist=False)
ax.set_axis_off()

# Boxplot (centre)
ax = fig.add_subplot(gs[0, 1])
# Makes the order of the order of the plot the same as the dendrogram
plot_order = groups.iloc[d_data['leaves']].index
p_data = mgkit.plots.boxplot.boxplot_dataframe(
    df,          # the whole dataframe, but
    plot_order,  # but only the taxa in plot_order are plotted
    ax,          # axes used
    box_vert=False,  # Make horizontal boxplots
    data_colours=plot_colors,  # colors assigned for each genus
    colours=dict(medians='k'),  # the median value is plot in black instead of the white default
    fill_box=False,  # doesn't fill the box
    fonts=dict(fontsize=16)
)
# changes the limit on the left to adjust the plot
ax.set_xlim(left=-.01)
# adds the labels for each genus
for index, taxon_id in enumerate(plot_order):
    ax.text(-0., index + 1, taxonomy[taxon_id].s_name.capitalize(), fontsize=20, 
            va='bottom', fontstyle='italic', fontweight='bold')

# adds all values to each boxplot
mgkit.plots.boxplot.add_values_to_boxplot(
    df, 
    ax, 
    p_data, 
    plot_order, 
    data_colours=plot_colors, 
    s=200
)
# changes the grid to only horizontal lines
ax.grid(which='major', axis='x')
# delete labels on the other axis
ax.set_yticklabels([])
ax.set_xlabel('pN/pS', fontsize=16)
# hides the spines
for spine in ax.spines.itervalues(): 
    spine.set_visible(False)

# Barchart (on the right)
ax = fig.add_subplot(gs[0, 2])
ax.barh(
    np.arange(0, len(plot_order), 1),
    gene_counts.loc[plot_order].values,
    align='center',  # aigns the bar on the tick
    height=0.8, # the "width" of the bar
    # the colours for each bar, must be in the same order
    color=[plot_colors[taxon_id] for taxon_id in plot_order]
)
# delete labels on the Y axis
ax.set_yticks([])
# tweaks the limits
ax.set_ylim(bottom=-0.5, top=len(plot_order) - 0.5)
ax.set_xlim(right=1100)
# hides all spines
for spine in ax.spines.itervalues():
    spine.set_visible(False)
# Sets the label and ticks position on the top
ax.set_xlabel('Number of Genes', fontsize=18)
ax.xaxis.set_label_position('top')
ax.xaxis.set_ticks_position('top')
for text in ax.get_xticklabels():
    text.set_fontsize(16)

# Save figure
fig.savefig('Tree_Genera-min{}-genes.pdf'.format(min_genes), bbox_inches='tight')
```

In [14]:

```
# For genera with at least 4 genes, print their mean pN/pS,
# only the 10 with the highest mean
for k, v in df.loc[gene_counts[gene_counts > 3].index].mean(axis=1).sort(ascending=False, inplace=False)[:10].iterkv(): print taxonomy[k].s_name.capitalize(), v
```

```
Chloroflexus 4.080022799
Thermococcus 2.93503497853
Robiginitalea 2.72562936457
Acinetobacter 2.01118153356
Riemerella 1.58645671387
Megasphaera 1.52161610603
Methanoculleus 1.09209103191
Sebaldella 1.01377024624
Staphylothermus 0.994292505742
Maribacter 0.93209472273
```

```
/Users/frubino/Dev/mgkit/dev-env/lib/python2.7/site-packages/ipykernel/__main__.py:3: FutureWarning: sort is deprecated, use sort_values(inplace=True) for for INPLACE sorting
  app.launch_new_instance()
/Users/frubino/Dev/mgkit/dev-env/lib/python2.7/site-packages/ipykernel/__main__.py:3: FutureWarning: iterkv is deprecated and will be removed in a future release, use ``iteritems`` instead.
  app.launch_new_instance()
```

In [15]:

```
# Latex table
df.index.name = 'Genus'
df.loc[taxon_ids].rename(
    # Adds emphasis on the genera names
    index={x: "\em{%s}" % taxonomy[x].s_name.capitalize() for x in df.index},
    # Escape sample names "_"
    # it is needed because escape is set to False (to keep the \em{})
    columns=lambda x: x.replace('_', '\_')
).to_latex(
    'taxa-variation.tex', 
    na_rep='', # NaN are empty string (duplicated in float_format)
    longtable=False, # A longtable in latex spans multiple pages, 
                     # but it's not possible to resize it by enclosing 
                     # it into \resizebox{\textwidth}{!}{ %table here% }
    # Uses just 2 digits after 0. NaN are represented as empty strings
    float_format=lambda x: u'{:.2}'.format(x if np.isfinite(x) else ''),
    # Manually escape special characters
    escape=False
)
```

In [16]:

```
# Excel table
df.loc[taxon_ids].rename(
    # Adds emphasis on the genera names
    index=lambda x: taxonomy[x].s_name.capitalize(),
).to_excel(
    'taxa-variation.xlsx',
    float_format='%.2f'
)
```

## Boxplot Top100 Genera by Median (min 4 genes)¶

In [17]:

```
fig, ax = mgkit.plots.get_single_figure(figsize=(10, 40), dpi=300)

# Patches for the legend
patches = [
    mpatches.Patch(color=sns.color_palette('Set1', 4)[2], label='Archaea'),
    mpatches.Patch(color=sns.color_palette('Set1', 4)[1], label='Bacteria'),
    mpatches.Patch(color=sns.color_palette('Set1', 4)[0], label='Fungi'),
    mpatches.Patch(color=sns.color_palette('Set1', 4)[3], label='Protozoa'),
]

# Plot order:
# 1 - use only genera with at least 4 genes
# 2 - sort by median value, in ascending order
plot_order = df.loc[
    gene_counts[gene_counts > 3].index
].median(axis=1).sort(ascending=True, inplace=False).index

# Plots only the top 100
plot_order = plot_order[-100:]
p_data = mgkit.plots.boxplot.boxplot_dataframe(
    df, 
    plot_order, 
    ax, 
    box_vert=False,  # horizontal boxplot
    # gets the taxa colours
    data_colours=get_ancestor_color(taxonomy, plot_order),
    # fills the boxes
    fill_box=True,
    fonts=dict(fontsize=12, rotation='horizontal'),
    # makes a dictiorny for the labels
    label_map={x: taxonomy[x].s_name.capitalize() for x in plot_order},
)

# Shows the label (uses the patches) and sets the
# location at the bottom right
_ = ax.legend(handles=patches, fontsize=16, loc=4)

for text in ax.get_yticklabels():
    text.set_style('italic')
    text.set_weight('bold')

# Changes labels
ax.set_xlabel('pN/pS', fontsize=16)
ax.set_ylabel('Genera', fontsize=16)
# Changes the limit
ax.set_xlim(right=7)
# Shows only the ticks for pN/pS
ax.grid(axis='y')

# Saves the figure
fig.tight_layout()
fig.savefig('boxplot-all-genera.pdf', bbox_inches='tight')
```

```
/Users/frubino/Dev/mgkit/dev-env/lib/python2.7/site-packages/ipykernel/__main__.py:16: FutureWarning: sort is deprecated, use sort_values(inplace=True) for for INPLACE sorting
```
